# Bi- and tri-valent T cell engagers deplete tumour-associated macrophages in cancer patient samples

**DOI:** 10.1186/s40425-019-0807-6

**Published:** 2019-11-21

**Authors:** Eleanor M. Scott, Egon J. Jacobus, Brian Lyons, Sally Frost, Joshua D. Freedman, Arthur Dyer, Hena Khalique, William K. Taverner, Alison Carr, Brian R. Champion, Kerry D. Fisher, Len W. Seymour, Margaret R. Duffy

**Affiliations:** 1 Department of Oncology, University of Oxford, Oxford, OX3 7DQ UK; 2 Churchill Hospital, Oxford University Hospital NHS Trust, Oxford, OX3 7LE UK; 3 PsiOxus Therapeutics Ltd., Abingdon, OX14 3YS UK

**Keywords:** Bispecific T cell engagers, Tumour-associated macrophages, Oncolytic virus, Tumour microenvironment

## Abstract

**Background:**

Tumour-associated macrophages (TAMs) are often implicated in cancer progression but can also exert anti-tumour activities. Selective eradication of cancer-promoting (M2-like) TAM subsets is a highly sought-after goal. Here, we have devised a novel strategy to achieve selective TAM depletion, involving the use of T cell engagers to direct endogenous T cell cytotoxicity towards specific M2-like TAMs. To avoid “on-target off-tumour” toxicities, we have explored localising expression of the T cell engagers to the tumour with enadenotucirev (EnAd), an oncolytic adenovirus in Phase I/II clinical trials.

**Method:**

A panel of bi- and tri-valent T cell engagers (BiTEs/TriTEs) was constructed, recognising CD3ε on T cells and CD206 or folate receptor β (FRβ) on M2-like macrophages. Initial characterisation of BiTE/TriTE activity and specificity was performed with M1- and M2-polarised monocyte-derived macrophages and autologous lymphocytes from healthy human peripheral blood donors. T cell engagers were inserted into the genome of EnAd, and oncolytic activity and BiTE secretion assessed with DLD-1 tumour cells. Clinically-relevant ex vivo models (whole malignant ascites from cancer patients) were employed to assess the efficacies of the free- and virally-encoded T cell engagers.

**Results:**

T cells activated by the CD206- and FRβ-targeting BiTEs/TriTEs preferentially killed M2- over M1-polarised autologous macrophages, with EC_50_ values in the nanomolar range. A TriTE with bivalent CD3ε binding – the first of its kind – demonstrated enhanced potency whilst retaining target cell selectivity, whereas a CD28-containing TriTE elicited non-specific T cell activation. In immunosuppressive malignant ascites, both free and EnAd-encoded T cell engagers triggered endogenous T cell activation and IFN-γ production, leading to increased T cell numbers and depletion of CD11b^+^CD64^+^ ascites macrophages. Strikingly, surviving macrophages exhibited a general increase in M1 marker expression, suggesting microenvironmental repolarisation towards a pro-inflammatory state.

**Conclusions:**

This study is the first to achieve selective depletion of specific M2-like macrophage subsets, opening the possibility of eradicating cancer-supporting TAMs whilst sparing those with anti-tumour potential. Targeted TAM depletion with T cell engager-armed EnAd offers a powerful therapeutic approach combining direct cancer cell cytotoxicity with reversal of immune suppression.

## Background

Immunosuppressive stromal cells represent critical obstacles to the success of cancer immunotherapy [1]. Key among these are tumour associated macrophages (TAMs), a diverse population of immune cells which promote angiogenesis, metastasis and immunosuppression [2]. Most TAMs resemble M2-polarised macrophages, with tissue-healing and/or immunoregulatory characteristics. Nevertheless, TAMs can also display features of M1-polarised macrophages, which carry out tumouricidal functions and promote T helper 1 immune responses. Consequently, the ratio of M2-to M1-polarised TAMs, and not total TAM numbers, is often an indicator of poor patient prognosis [3–9].

TAM-targeting therapeutic strategies include: i) systemic macrophage depletion (e.g. with bisphosphonates [10]), ii) inhibition of macrophage precursor recruitment/differentiation (e.g. with CSF-1/CSF-1R inhibitors [11]), and iii) repolarisation of macrophages towards an M1-like phenotype (e.g. with CD40 agonists [12]). Several TAM-targeting agents are under clinical evaluation [13]. One of the most advanced, pexidartinib, is a CSF-1R inhibitor which demonstrated impressive results in a Phase III clinical trial in patients with CSF-1-driven giant cell tumours [14], and is undergoing Phase II trials for other solid tumours [15].

Whilst promising, many of these approaches fail to address the heterogeneity of TAMs, which, beyond the extremes of M1/M2-polarisation, likely exist as subsubsets exerting distinct functions [16]. Selectively depleting cancer-supporting TAM subsets, whilst sparing those with anti-tumour potential, is highly desirable.

Bispecific T cell engagers (BiTEs) redirect endogenous T cells to target cells. Derived from two single chain variable fragments (scFv), BiTEs recognise CD3ε and a chosen target antigen [17]. A CD19-targeting BiTE, blinatumomab, is approved for the treatment of relapsed/refractory B-cell precursor acute lymphoblastic leukaemia [18], while BiTEs recognising solid tumour-associated antigens are under pre-clinical or clinical evaluation [19]. Promising pre-clinical results have also been obtained with a BiTE recognising cancer-associated fibroblasts (via fibroblast activation protein (FAP)) [20, 21]. “On-target off-tumour” side effects of BiTEs are of particular concern when targeting stromal cells; however, these may be avoided by localising BiTE expression to tumours with engineered oncolytic viruses (OVs) [20–23]. The FAP BiTE was particularly effective when expressed by an OV, proving this to be a synergistic regime for co-targeting malignant and stromal cells [20, 21]. However, a similar approach to deplete TAMs is yet to be realised.

One variation between different TAM subsets that may be exploited with BiTEs is their differential expression levels of certain surface markers, such as CD206 and folate receptor (FR)β. In mouse mammary tumours, CD206^+^ TAMs were major histocompatibility complex (MHC)-II^low^ and more angiogenic than their CD206^−^ counterparts [24]. For ovarian and hepatocellular carcinomas, the density of CD206^+^ TAMs, and not overall CD68^+^ TAM density, was identified as a poor prognostic factor [5, 25]. In pancreatic cancer patients, a high number of FRβ^+^ TAMs correlated with increased metastasis and poor prognosis, with FRβ^+^ TAMs being VEGF^+^ [26]. In melanoma and breast adenocarcinoma patient samples, FRβ^+^ TAMs were CD163^+^ and IL-10-producing, suggesting that they represent an immunosuppressive TAM population [27].

Here, we explore approaches to redirect T cell toxicity towards cancer-supporting TAMs. We report the development of novel bi- and tri-valent T cell engagers targeting CD206 and FRβ, and have evaluated their therapeutic potential in conjunction with OVs. Using clinically-relevant patient samples, we demonstrate activation of endogenous T cells by free and virally-expressed TAM-targeting T cell engagers, leading to endogenous macrophage depletion. This study marks the first to achieve T cell redirection towards macrophages with T cell engagers, and highlights the potential of this approach as a means of selectively targeting cancer-promoting TAMs.

## Methods

### Cell lines and maintenance

HEK293A, A549 and DLD-1 (ATCC) were maintained in Dulbecco’s modified Eagle medium (DMEM, Sigma-Aldrich, UK) supplemented with 10% (v/v) heat-inactivated foetal bovine serum (FBS, Gibco, UK). Transfections were performed in Opti-MEM (Gibco, UK), whilst virus infections were carried out using DMEM with 2% (v/v) FBS. Primary cells were maintained in X-VIVO 10 (Lonza, UK) with 1% (v/v) heat-inactivated human serum (HS, Sigma-Aldrich, UK). Cells were grown at 37 °C, 5% CO_2_ and 95% humidity.

### Isolation of lymphocytes and monocytes from peripheral blood

Human peripheral blood from anonymised healthy donors was obtained from the NHS Blood and Transfusion Service (Oxford, UK). PBS-diluted blood was overlaid onto Ficoll-Paque Plus (GE Healthcare, UK), then centrifuged (950 g, 30 min, RT). PBMCs were collected and washed with PBS, then resuspended in RPMI-1640 medium supplemented with 10% FBS. Cells were overlaid onto Percoll PLUS (46% in RPMI-1640, 10% FBS, 285 mOsm; GE Healthcare, UK) and centrifuged as before. The monocyte and lymphocyte fractions (the interphase and pellet, respectively) were collected and washed with PBS.

### Monocyte-derived macrophage (MDM) generation and polarisation

Monocytes were differentiated into macrophages through 6 days’ culture in medium containing 1% HS. Where specified, day-4 MDMs were polarised for 48 h using IL-4 (25 ng/mL, Miltenyi Biotec, UK, #130–095-373), IL-6 (25 ng/mL, Miltenyi Biotec, UK, #130–095-365) or IFN-γ (25 ng/mL, Miltenyi Biotec, UK, #130–096-873) and LPS (10 ng/mL, Sigma-Aldrich, UK). To generate FRβ^high^ MDMs, HS was omitted and monocytes were instead differentiated with recombinant M-CSF (50 ng/mL, Miltenyi Biotec, UK, #130–096-491). Where specified, monocytes were differentiated in the presence of ascites fluid (50% v/v).

### Malignant ascites processing and characterisation

Ascites samples were acquired with informed consent from routine drainage of cancer patients at the Churchill Hospital, Oxford, UK. Ascites were centrifuged (400 g for 10 min at room temperature) to separate the cellular and fluid components. The fluid was stored at − 20 °C until required. The cellular fraction was treated with red blood cell lysis buffer (Qiagen, UK, #158904) and cryopreserved until further use. For characterisation, cells were stained with Live/Dead Fixable Near IR stain (Invitrogen, UK, #L10119) and antibodies targeting CD4 (OKT4, Biolegend, UK, #317416), CD8 (HIT8a, Biolegend, UK, #300912), EpCAM (9C4, Biolegend, UK, #324206), FAP (427,819, R&D Systems UK, #MAB3715), PD-L1 (MIH3, Biolegend, UK, #374512), CD11b (ICRF44, Biolegend, UK, #301310), CD206 (15–2, Biolegend, UK, #321106) and folate receptor β (94b/FOLR2, Biolegend, UK, #391704), then analysed by flow cytometry using an Attune™ NxT Flow Cytometer (Thermo Fisher, UK).

### Engineering and production of T cell engagers

BiTEs were generated by joining, with a glycine-serine linker, a single chain variable fragment (scFv) specific for CD3ε (L2K, patent #WO2004/106380) to a CD206-targeting nanobody (NbhMMRm3.1, patent #WO2014/140376Al) or a folate receptor β-targeting scFv (m923, patent #US2016/0207999A1). TriTEs were engineered similarly, with an anti-CD28 scFv (Clone 9.3, publically available on the ENA database, #AJ507107.1) or a second anti-CD3 scFv (L2K, patent #WO2004/106380) added with a glycine-serine linker to the N-terminus of the parental BiTE. Control BiTEs/TriTEs contained antibody fragments targeting irrelevant antigens. BiTEs/TriTEs contained an immunoglobulin signal peptide at the N-terminus for mammalian secretion and a deca-histidine (His) tag at the C-terminus for detection/ quantification. Using HiFi Master Mix (NEB, UK) to perform Gibson assembly [28], DNA fragments were inserted into an expression vector (pSF-CMV-Amp, Oxford Genetics Ltd., UK), under the control of a CMV promoter. Transgene insertion was confirmed by restriction digest and Sanger sequencing (Eurofins Genomics, Germany).

BiTE/TriTE-containing supernatants were produced through transfection of HEK293A using Lipofectamine 2000 (Invitrogen, UK) at a DNA:Lipofectamine ratio of 1:3 (w/v). Supernatants were harvested 48 h post-transfection and centrifuged to remove cellular components (400 g, 10 min, RT), then concentrated with Amicon Ultra-15 Centrifugal Filter Units (Merck, UK). Concentrated BiTE/TriTE-containing supernatants were aliquoted and stored at − 80 °C.

### Generation of T cell engager-expressing enadenotucirev

BiTEs/TriTE-encoding transgenes were inserted into a parental EnAd plasmid (EnAd2.4) using HiFi Master Mix (NEB, UK). Transgene cassettes contained a CMV promoter to drive BiTE/TriTE expression and a 3′ polyadenylation sequence. Successful transgene insertion was confirmed by restriction digest/Sanger sequencing (Eurofins Genomics, Germany). EnAd-CMV-BiTE/TriTE constructs were linearised with AscI (NEB, UK) and transfected into HEK293A cells using Lipofectamine 2000 (Invitrogen, UK). Cells and supernatant were harvested upon observation of cytopathic effect. Single virus clones were isolated by plaque-purification, then amplified and purified by double caesium chloride banding [29]. Virus stocks were titred by PicoGreen (Life Technologies, UK), yielding estimates of virus particles (vp)/mL (Additional file 13). For quality control, all viruses were analysed by anion exchange HPLC (Shimadzu Prominence, Japan), using GMP-grade EnAd (provided by PsiOxus Therapeutics, UK) to generate a standard curve.

### Celigo-based cytotoxicity assay

MDM killing was assessed using Celigo-based image cytometry (Nexcelom Bioscience, USA). Day-6 MDMs were harvested, stained with carboxyfluorescein succinimidyl ester (CFSE, Invitrogen, UK, #C34554) and seeded at 25,000 cells/well into 96-well plates. The next day, MDMs were treated with BiTE/TriTEs, in the presence/absence of autologous lymphocytes (E:T ratio of 10:1, unless otherwise specified). In some experiments, co-cultures included 50% ascites fluid. Four days later, lymphocytes were removed and MDMs stained with propidium iodide (PI; diluted to 1 μg/mL in PBS, Sigma-Aldrich, UK, #P4864), and imaged on a Celigo image cytometer. Live MDMs were identified as being both CFSE-positive and PI-negative. % Live cells was calculated as follows:

$$ \% Live\ cells=\frac{\ {CFSE}^{+}{PI}^{-}\  count\ (test)}{CFSE^{+}{PI}^{-}\  count\ (mock)}\times 100\% $$

### Characterisation of T cell activation

T cell activation was determined by flow cytometric analysis of CD25 expression. Lymphocytes were incubated in 96-well plates with/without autologous target MDMs (E:T ratio of 10:1, unless otherwise specified), and treated with BiTEs/TriTEs. Some experiments were conducted in 50% ascites fluid. After 4 days’ co-culture, lymphocytes were harvested and stained with anti-CD4 (OKT4, Biolegend, UK, #317416), −CD8 (HIT8a, Biolegend, UK, #300912), −CD25 (BC96, Biolegend, UK, #302606), −CD69 (FN50, Biolegend, UK, # 310904), −CD107a (H4A3, Biolegend, UK, #328608) and -HLA-DR (L243, Biolegend, UK, #307604) antibodies.

### Ex vivo experiments

Unpurified ascites cells were seeded at 200,000 cells/well in 100 μL medium into flat-bottom low-adherence 96-well plates. After resting overnight, cells were treated with BiTEs/TriTEs or viruses, diluted in 100 μL medium or autologous ascites fluid. Five days later, cells were harvested and processed for flow cytometry, using Live/Dead Fixable Near IR stain (Invitrogen, UK, #L10119) and anti-CD11b (ICRF44, Biolegend, UK, #301310), −CD64 (10.1, Biolegend, UK, #305006), −CD86 (IT2.2, Biolegend, UK, #305422), −CD80 (2D10, Biolegend, UK, #305208), −CD4 (OKT4, Biolegend, UK, #317408), −CD8 (HIT8a, Biolegend, UK, #300912) and -CD25 antibodies (BC96, Biolegend, UK, #302606).

### Enzyme-linked immunosorbent assay (ELISA)

ELISAs were performed with commercially-available kits measuring IL-6 (Biolegend, UK, #30504), IL-10 (Biolegend, UK, #430604), IFN-γ (Biolegend, UK, #430104), TGF beta-1 Human/Mouse ELISA Kit (Invitrogen, UK, #88–8350-88) and CD206 (RayBiotech, USA, #ELH-MMR-1), following manufacturers’ instructions. Prior to analysis, samples were centrifuged (400 g, 10 min, RT) to remove cellular components and diluted two-fold (IL-6 and IL-10), five-fold (TGF-β), ten-fold (IFN-γ) or thirty-fold (CD206) in PBS.

### Multiplex immunoassay

Cytokines and chemokines in ascites cell supernatants were quantified using the LEGENDplex Human Macrophage/Microglia panel kit (Biolegend, UK, #740526) and flow cytometric analysis, according to manufacturers’ instructions.

### Immunoblotting

BiTE/TriTEs were detected by immunoblotting with anti-C-terminal-His antibody (3D5, Invitrogen, UK, #R930–25). For western blotting analysis, supernatants were fractionated by SDS-PAGE and transferred to a nitrocellulose membrane. For dot blot analysis (Additional file 1), two-fold serial dilutions of the supernatants were applied directly to a nitrocellulose membrane. To generate a standard curve, a deca-His-tagged standard protein of known concentration was serially-diluted and applied in parallel. Membranes were probed with an anti-C-terminal-His tag (1:5000, clone 3D5, Invitrogen, UK, #46–069) primary antibody, then a horseradish peroxidase (HRP)-conjugated anti-mouse secondary antibody (1:3000, Cell Signalling Technology, UK, #7076). SuperSignal West Dura Extended Duration Substrate (Thermo Fisher, UK, #34075) was applied and the membrane exposed to X-ray film, which was developed in an automatic film processor (Agfa CP1000).

### Statistical analysis

Statistical analysis was performed using one- or two-way ANOVA tests, with Dunnett or Bonferroni post hoc analysis, respectively. All data are presented as mean ± SD. The significance levels used were *P* = 0.01–0.05 (*), 0.001–0.01 (**), 0.0001–0.001 (***). Experiments were performed in biological triplicate, unless otherwise stated.

## Results

### CD206 and folate receptor β constitute markers of human TAMs

**Fig. 1:**
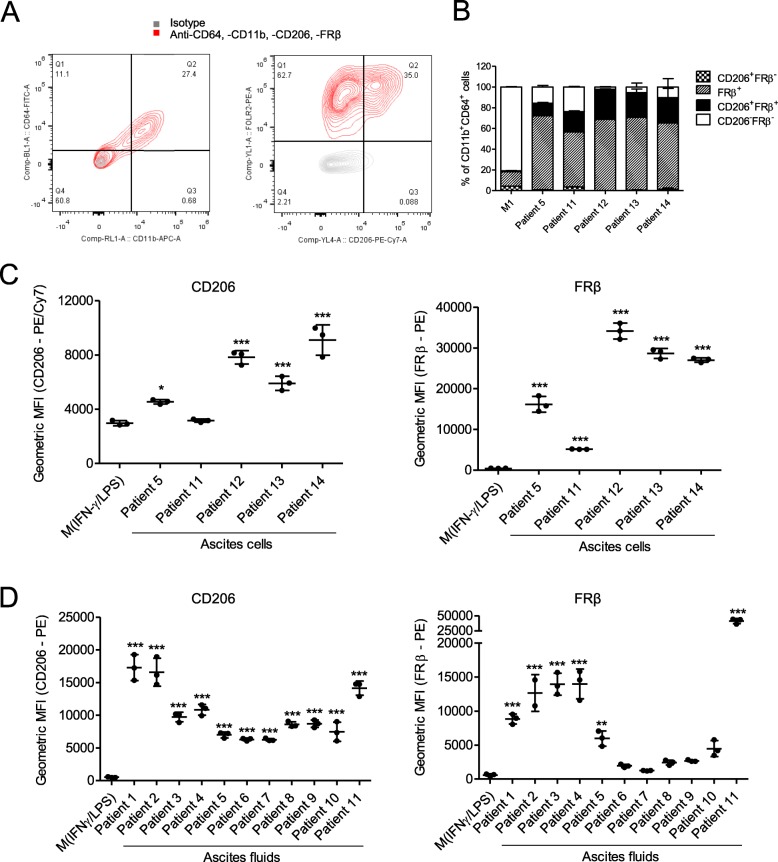
CD206 and folate receptor (FR) β are markers of human tumour-associated macrophages. **a** Representative flow cytometry plots showing CD206 and FRβ expression on primary ascites cells after gating for CD11b^+^/CD64^+^ double positivity. **b**, **c,** Percentage positivity for (**b**) and expression levels of (**c**) CD206 (PE/Cy7) and FRβ (PE) on primary CD11b^+^/CD64^+^ ascites cells from five different cancer patients, and on monocyte-derived macrophages (from healthy donors) polarised with 10 ng/mL LPS and 25 ng/mL IFN-γ (“M(IFN-γ/LPS)”), as determined by flow cytometric analysis. **d** Primary human monocytes from healthy donors were differentiated into macrophages through 6 days’ culture in medium containing 1% human serum. Where indicated, culture medium contained 10 ng/mL LPS and 25 ng/mL IFN-γ (“M(IFN-γ/LPS)”, added on Day 4), or 50% acellular ascitic supernatant from 11 different patients (“Patient 1″-“Patient 11″, added on Day 0). Expression levels of CD206 (PE) and FRβ (PE) were determined by flow cytometry. **c**, **d** Data show mean ± SD of biological triplicates. Statistical analysis was performed by one-way ANOVA with Dunnett’s post-hoc analysis compared with “M(IFN-γ/LPS)” (*, *P* < 0.05; **, *P* < 0.01; ***, *P* < 0.001)

We first determined the expression of two M2-like macrophage markers, CD206 and FRβ, in clinically-relevant models of human TAMs. Primary ascites cells from five cancer patients were characterised by flow cytometry (Fig. 1a). Ascites-associated macrophages (identified as being CD11b^+^CD64^+^) expressed CD206 (4/5 patients) and FRβ (5/5 patients) at higher levels than M1-polarised monocyte-derived macrophages (MDMs; derived from healthy peripheral blood mononuclear cells (PBMCs)) (Fig. 1b and c). In an alternative approach, we cultured PBMC-derived human monocytes with cell-free malignant ascites fluid, which reportedly generates MDMs that resemble human TAMs [30]. Using ascites fluid from 11 cancer patients (Additional file 15), we observed significant up-regulation of CD206 (11/11 patients) and FRβ (6/11 patients) on human PBMC-derived MDMs, as compared to M1-polarised MDMs (Fig. 1d).

### Generation and characterisation of TAM-targeting BiTEs

**Fig. 2:**
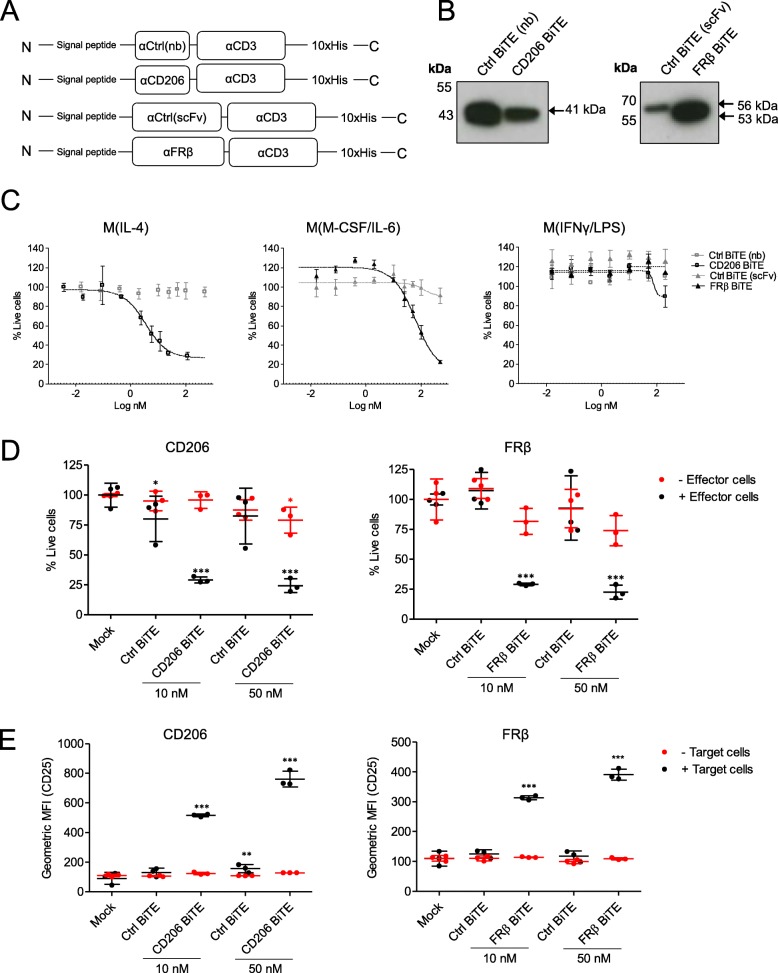
CD206- and FRβ-targeting BiTEs activate primary human T cells to kill autologous M2-polarised macrophages. **a** Schematic representations of CD206- and FRβ-targeting BiTEs. **b,** Western blot analysis of supernatants from HEK293A cells 48 h after transfection with BiTE expression plasmids. Blots were probed with a mouse anti-His primary antibody, followed by an HRP-conjugated anti-mouse secondary antibody. **c** Human MDMs were polarised as indicated, stained with CFSE, and treated with T cells (10:1 E:T ratio) and increasing concentrations of BiTEs. Macrophage killing was assessed 96 h later by propidium iodide staining and Celigo image cytometry. **d** MDMs were stained with CFSE and treated with the indicated concentrations of BiTE in the presence or absence of T cells (10:1 E:T ratio). 96 h later, cytotoxicity was assessed by propidium iodide staining and analysis with a Celigo image cytometer. **e** T cell activation in the presence or absence of target cells was assessed by flow cytometric measurement of CD25 expression 96 h after BiTE addition. Data show mean ± SD of biological triplicates (**c**, **d** and **e**). Statistical analysis was performed by two-way ANOVA with Bonferroni post-hoc tests comparing with the relevant “Mock” condition (**d** and **e**) (*, *P* < 0.05; **, *P* < 0.01; ***, *P* < 0.001)

Human TAM-targeting BiTEs were engineered by joining, with a glycine-serine (GGGGS) linker, a single chain variable fragment (scFv) specific for CD3ε to domains specific for CD206 and FRβ (Fig. 2a). The CD206-binding domain was a nanobody, whilst the FRβ-binding domain was a scFv. Antibody fragments were selected based on their similarly high affinities (K_D_ of 3.4 nM and 2.48 nM for CD206-binding and FRβ-binding fragments, respectively [31], patent #WO2014/140376Al). Control (Ctrl) BiTEs, with the same CD3ε-binding domain and a nanobody or scFv recognising irrelevant antigens (rabies virus protein for the CD206 BiTE and filamentous hemagglutinin adhesin of *Bordetella pertussis* for the FRβ BiTE, respectively), were also generated. BiTEs contained a signal peptide for secretion and a deca-histidine tag for detection. BiTE constructs were cloned into expression vectors under the control of the cytomegalovirus immediate early (CMV) promoter. All BiTEs were expressed and secreted following transfection of HEK293A cells (Fig. 2b).

Dose-responses were performed using PBMC-derived human lymphocytes and autologous MDMs, which were M2-polarised with IL-4 or M-CSF/IL-6, generating CD206- or FRβ-high target cells, respectively (Additional file 2). Other MDMs were M1-polarised with IFN-γ/LPS, giving low levels of CD206 and FRβ expression (Additional file 2). Both BiTEs triggered T cell-mediated toxicity towards M2-polarised MDMs, with nanomolar EC_50_ values (CD206 BiTE, 3.4 nM; FRβ BiTE, 61.22 nM) (Fig. 2c). There was no killing of M1-polarised MDMs at any concentration of FRβ BiTE, and only minor cytotoxicity at the highest dose of the CD206 BiTE (Fig. 2c). BiTE-mediated cytotoxicity was strictly dependent on the presence of lymphocytes (Fig. 2d). Likewise, T cell activation (as assessed by CD25, CD69, HLA-DR and CD107a expression) was observed only upon co-culture with target cells (Fig. 2e and Additional file 3).

Consistent with previous work concerning cancer cell-targeting BiTEs [32], FRβ and CD206 BiTE-induced T cell-mediated killing of macrophages was dependent on perforin and not death receptor pathways, with a significant decline in BiTE-mediated MDM cytotoxicity upon addition of a perforin inhibitor, concanamycin A, but not inhibitors of Fas/FasL or TRAIL (Additional file 4).

### Activity of TAM-targeting BiTEs in the presence of malignant ascites fluids

**Fig. 3:**
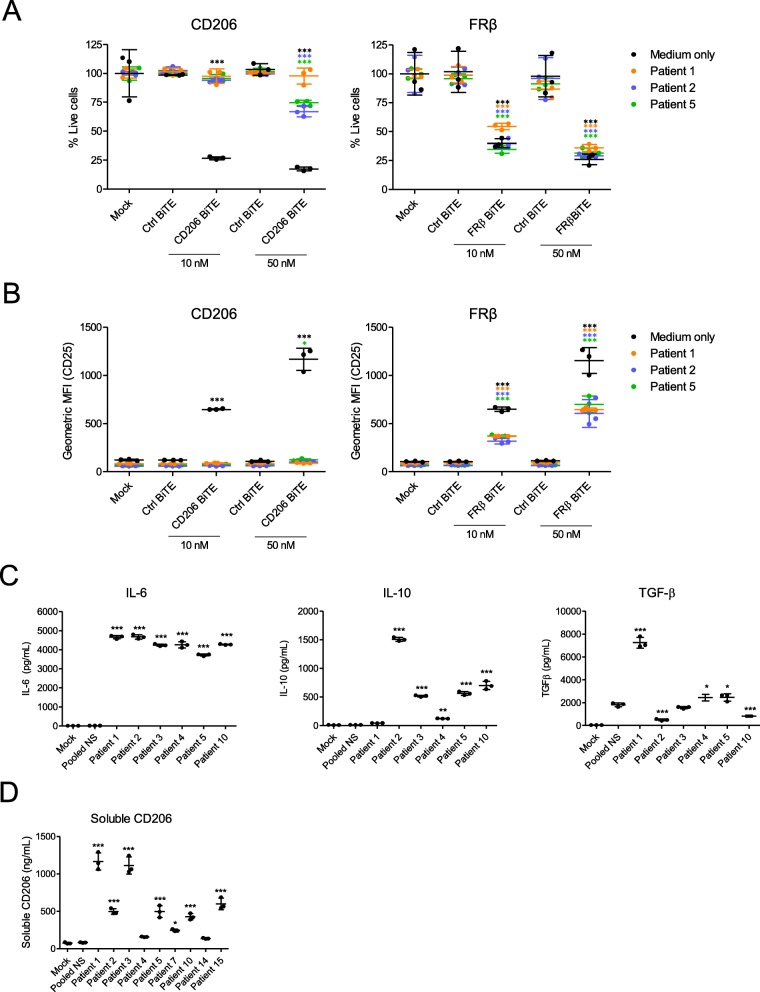
Human malignant ascites supernatant suppresses CD206 BiTE activity but not FRβ BiTE activity. **a** CFSE-stained MDMs were co-cultured with T cells (10:1 E:T ratio) and the indicated BiTEs, in the presence or absence of 50% ascitic supernatant from three patients (Patients 1, 2 and 5). 96 h later, percentage live MDMs was determined with propidium iodide staining and a Celigo image cytometer. **b** T cell activation was assessed by flow cytometric analysis of CD25 expression after 96 h of co-culture with MDMs and the indicated BiTEs, in the presence or absence of 50% ascitic supernatant from three patients (Patients 1, 2 and 5). **c** Quantities of IL-6, IL-10 and total (active and latent) TGF-β in malignant ascites fluid from six different patients, as determined by enzyme-linked immunosorbent assay. Normal serum (NS) pooled from three healthy donors was included as a control. **d** Quantities of soluble CD206 in malignant ascites fluid from nine different patients was determined by enzyme-linked immunosorbent assay. Pooled NS was used as a control. Each condition was measured in biological triplicate and represented as mean ± SD (**a**-**d**). Statistical significance was assessed by one-way ANOVA with Dunnett’s post-hoc analysis compared with “Pooled NS” (**c**, **d**), or two-way ANOVA followed by Bonferroni post-hoc analysis, with each treatment being compared to the “Mock” condition within the relevant group (**a** and **b**). (*, *P* < 0.05; **, *P* < 0.01; ***, *P* < 0.001; ns, non-significant)

We next asked whether the TAM-targeting BiTEs would retain their activity in acellular malignant ascites, which is rich in soluble immunoregulatory factors [33]. Using human MDMs and autologous lymphocytes from healthy peripheral blood, we performed BiTE cytotoxicity assays in the presence of ascites fluid (50% v/v) from three cancer patients (Fig. 3a and b). FRβ BiTE activity was largely unaffected, triggering robust T cell activation and cytotoxicity (Fig. 3a and b). The efficacy of the CD206 BiTE, however, was greatly diminished, with little or no T cell activity observed in ascites fluid (Fig. 3a and b). Elevated levels of three prominent immunomodulatory factors, IL-6, IL-10 and TGF-β, were observed in all ascites samples (Fig. 3c), relative to pooled healthy human serum. Furthermore, soluble CD206, which may block BiTE binding to membrane-bound CD206, was detected at high levels in most ascites fluids (Fig. 3d). Interestingly, the ascites sample with the greatest inhibitory effect (“Patient 1”) contained high levels of IL-10, TGF-β and soluble CD206, perhaps indicating important roles for these factors in limiting CD206 BiTE activity.

### Engineering a CD206-targeting trivalent T cell engager (TriTE) with increased potency

**Fig. 4:**
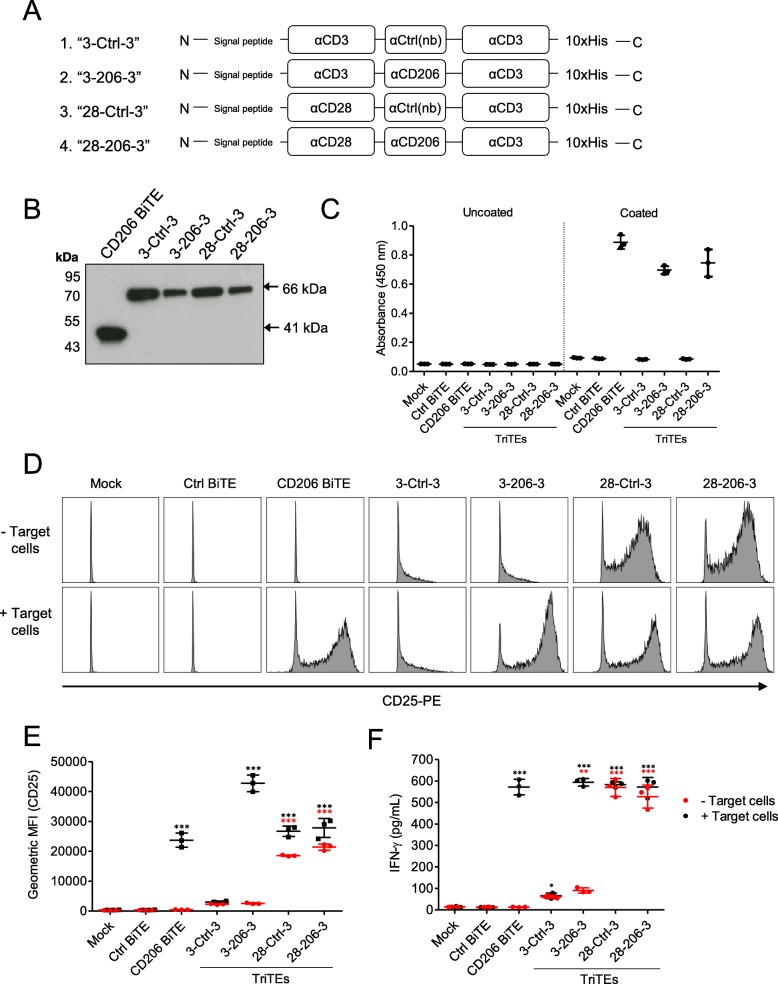
Addition of a second T cell-binding domain to the parental CD206 BiTE. **a** Schematic representations of CD206-targeting TriTEs. **b** Western blotting analysis of supernatants from HEK293A cells transfected with TriTE expression plasmids 48 h previously. Blots were probed with a mouse anti-His primary antibody and an HRP-conjugated anti-mouse secondary antibody. **c** BiTE and TriTE binding to recombinant CD206 protein, as determined by ELISA using a mouse anti-His primary antibody and an HRP-conjugated anti-mouse secondary antibody. **d**-**f** T cells were cultured for 96 h with the indicated BiTEs/TriTEs at a dose of 50 nM in the presence or absence of target cells. T cell activation was assessed by measuring CD25 expression by flow cytometry, with representative histograms displayed in (**d**) and geometric MFI values displayed in (**e**). IFN-γ levels in the supernatants were quantified by ELISA (**f**). Data show mean ± SD of biological triplicates (**c**, **e** and **f**). Statistical significance was assessed by two-way ANOVA followed by Bonferroni post-hoc analysis, with each treatment being compared to the “Mock” condition within the relevant group (**e** and **f**) (*, *P* < 0.05; **, *P* < 0.01; ***, *P* < 0.001)

T cell activation is determined by a balance between stimulatory and inhibitory signals. We asked whether adding a second T cell-activating domain to the N-terminus of the parental CD206 BiTE would push the balance towards T cell activation in ascites fluid. Two trivalent T cell engagers (TriTEs) were engineered; one contained a second anti-CD3-binding domain (referred to as “3–206-3”), whilst the other contained an anti-CD28 binding domain, providing T cell co-stimulation (“28–206-3”) (Fig. 4a). Matched Ctrl TriTEs were generated in parallel (“3-Ctrl-3”, “28-Ctrl-3”) (Fig. 4a). All four TriTEs were secreted by transfected HEK293A cells (Fig. 4b). CD206 binding was not compromised by the additional T cell-binding domains (Fig. 4c).

Interestingly, addition of an anti-CD28 domain to the parental BiTE resulted in non-specific T cell activation, with similar levels of CD25 expression induced by “28–206-3” and “28-Ctrl-3” TriTEs in both the presence and absence of target cells (Fig. 4d and e). These findings were confirmed by the observation of IFN-γ secretion by T cells treated with both “28–206-3” and “28-Ctrl-3” TriTEs, in the presence and absence of target cells (Fig. 4f).

Conversely, the CD206 TriTE with bivalent CD3 binding, “3–206-3”, triggered a significant (186-fold, Fig. 4e) rise in T cell CD25 expression only upon co-culture with target cells (Fig. 4d and e). Indeed, the relevant control TriTE, “3-Ctrl-3”, was unable to elicit significant T cell activation (Fig. 4d and e). Furthermore, the “3–206-3” TriTE caused an induction (43.5-fold) of IFN-γ secretion only in the presence of target cells, although a small rise (4.8-fold) was also observed when T cells were treated with the “3-Ctrl-3” TriTE (Fig. 4f), suggesting a degree of non-specific T cell activation by bivalent CD3 TriTEs.

### A CD206 TriTE with bivalent CD3 binding retains target cell selectivity and outperforms the parental BiTE in suppressive ascites fluid

**Fig. 5:**
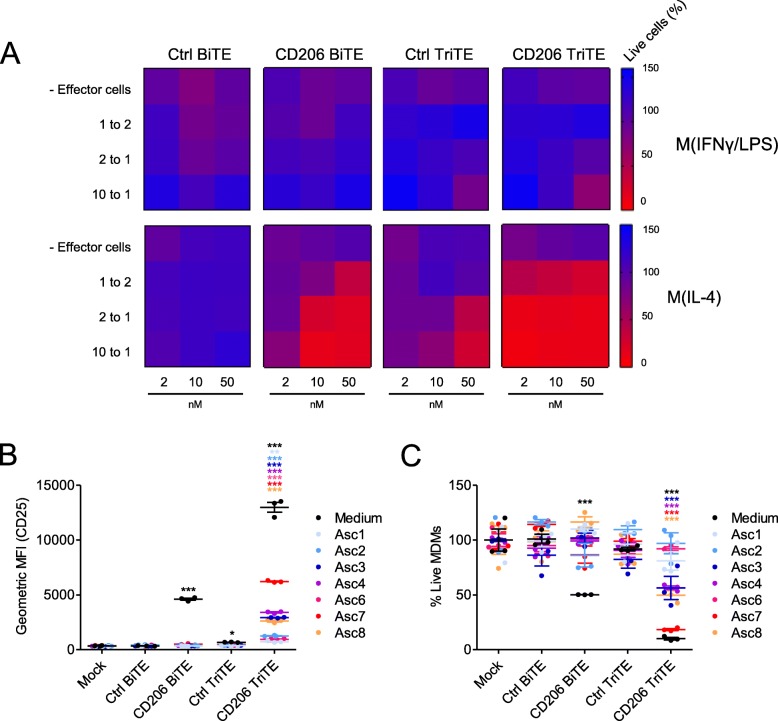
A CD206 TriTE with bivalent CD3 binding retains specificity for M2 macrophages and overcomes ascites suppression. **a** Monocyte-derived macrophages were polarised as indicated, CFSE-stained, and co-cultured for 96 h with T cells at increasing E:T ratios and BiTE/TriTE concentrations. % Live cells were calculated with propidium iodide staining and Celigo image cytometry, with values displayed as a heat map. **b** T cells were co-cultured with monocyte-derived macrophages and BiTEs/TriTEs in the presence or absence of 50% ascites fluid from seven different patients (Patients 1, 2, 3, 4, 6, 7 and 8). 96 h later, CD25 expression was determined by flow cytometry. **C,** CFSE-stained monocyte-derived macrophages were treated with T cells (10:1 E:T ratio) and BiTEs/TriTEs in medium alone or 50% ascites supernatant from seven different patients (Patients 1, 2, 3, 4, 6, 7 and 8). 96 h later, cells were stained with proprodium iodide and analysed with a Celigo image cytometer to calculate % live cells. Data show mean ± SD of biological triplicates (**b**, **c**). Statistical significance was assessed by two-way ANOVA followed by Bonferroni post-hoc analysis, with each treatment being compared to the relevant “Mock” condition (**b**, **c**) (*, *P* < 0.05; **, *P* < 0.01; ***, *P* < 0.001)

Given the lack of antigen dependency of the 28–206-3 TriTE, we proceeded only with the 3–206-3 TriTE (hereafter termed “CD206 TriTE”). We next determined its selectivity for M2 (IL-4-polarised) over M1 (IFN-γ/LPS-polarised) MDMs (Additional file 2). A checkerboard approach was employed, with MDMs subjected to increasing BiTE/TriTE concentrations in the presence of increasing numbers of T cells (i.e. greater effector:target (E:T) ratios). Cytotoxicity towards M2-polarised macrophages was observed even at E:T ratios (< 2:1) and BiTE/TriTE concentrations (< 10 nM) (Fig. 5a). Conversely, M1-polarised MDMs were killed only when E:T ratio and TriTE concentration were simultaneously high (E:T ratio of 10:1 and TriTE concentration of 50 nM, Fig. 5a). Similarly, the Ctrl TriTE only elicited macrophage cytotoxicity at high E:T and TriTE concentrations (Fig. 5a). Notably, the CD206 TriTE induced cytotoxicity of M2-polarised MDMs at lower concentrations and more physiologically-relevant E:T ratios than the CD206 BiTE (Fig. 5a). For instance, at a concentration of 2 nM and an E:T ratio of 2:1, the CD206 TriTE triggered a marked decrease in % live macrophages to 10.6%, whilst the CD206 BiTE was completely ineffective (Fig. 5a; Additional file 5).

Next, TriTE activity in the presence of an expanded panel of ascites fluids was assessed. At a low concentration of 10 nM, the CD206 TriTE triggered significant T cell activation in all ascites fluids tested, whilst the CD206 BiTE was ineffective (Fig. 5b). Superior cytotoxicity of the CD206 TriTE towards macrophages was also observed; in four of seven ascites fluids, the CD206 TriTE (at 10 nM) induced a robust decrease in % live macrophages (to 56.3, 56.5, 18.4 and 49.9% for Patients 3, 4, 7 and 8 respectively), while no significant cytotoxicity was exerted by the CD206 BiTE in the presence of the fluids (Fig. 5c).

### Optimised TAM-targeting T cell engagers deplete endogenous macrophages in whole malignant ascites

We then explored whether the FRβ-targeting BiTE may also be improved upon by molecular engineering. Unexpectedly, we found that reversing the order of scFv domains along the single chain (to N-αCD3-αFRβ-C) increased efficacy, without compromising target cell selectivity (Additional file 6). The new BiTE (“3FR BiTE”) exhibited an EC_50_ value of 10.63 nM – ≈6 times lower than that of the original BiTE (“FR3 BiTE”; EC_50_ of 61.22 nM) (Additional file 6). Both FRβ BiTE orientations were compared in the remaining experiments.

**Fig. 6:**
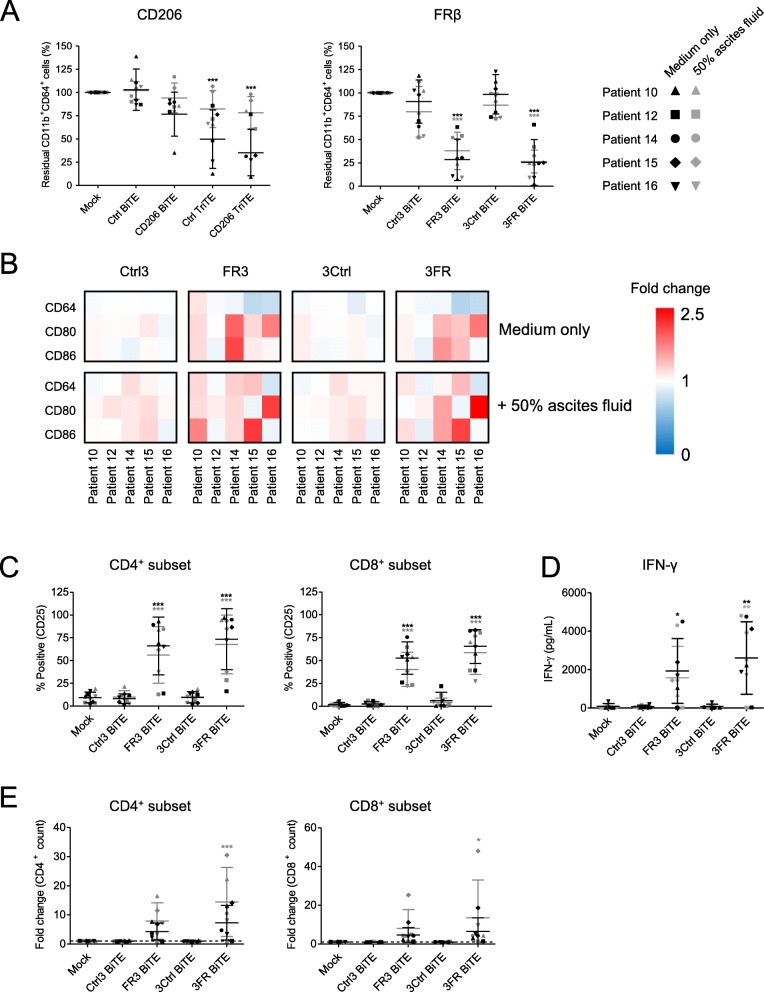
CD206- and FRβ-targeting T cell engagers activate endogenous ascites T cells to kill ascites macrophages. **a**-**e** Total unpurified ascites cells from five different patients were cultured for five days with 50 nM BiTEs/TriTEs in medium only or 50% ascites supernatant from the same patient sample. **a**, **b** Cells were stained with anti-CD11b, anti-CD64, anti-CD80 and anti-CD86 antibodies, as well as a LIVE/DEAD fixable stain, then analysed by flow cytometry. **a** % Live residual CD11b^+^CD64^+^ cells were calculated relative to “Mock”-treated samples. **b** Fold-changes in geometric MFI values of CD64, CD80 and CD86 on live CD11b^+^CD64^+^ ascites cells were calculated relative to “Mock”-treated samples for each patient sample. **c** Activation of endogenous CD4^+^ and CD8^+^ ascites T cells was assessed by flow cytometric measurement of CD25 expression. **d** IFN-γ levels in the culture supernatants were determined by ELISA. **e** Numbers of CD4^+^ and CD8^+^ cells were determined through addition of counting beads to samples immediately prior to antibody staining. Fold-changes in CD4^+^ and CD8^+^ cell count were calculated relative to “Mock”-treated samples. Data show the grand mean ± SD of five individual patient means (calculated from biological triplicate). Statistical significance was assessed by two-way ANOVA followed by Bonferroni post-hoc analysis, with each treatment being compared to the relevant “Mock” condition (**a**, **c**-**e**) (*, *P* < 0.05; **, *P* < 0.01; ***, *P* < 0.001)

Malignant ascites contains a mixture of tumour cells and cancer-associated fibroblasts, lymphocytes, myeloid-derived suppressor cells and M2-like macrophages, making it a valuable tumour-like model for studying BiTE efficacy. Ascites cells from five patients (characterisation in Additional files 7 and 14) were treated with the TAM-targeting T cell engagers in the presence or absence of autologous ascites fluid. The FRβ-targeting BiTEs triggered a marked depletion of ascites macrophages; in the presence of ascites fluid, % residual CD11b^+^CD64^+^ cells decreased to an average of 37.9 and 26.4% for the FR3 and 3FR BiTEs, respectively (Fig. 6a). CD206 TriTE treatment, however, induced killing of CD11b^+^CD64^+^ cells only in the absence of ascites fluids (Fig. 6a); furthermore, this activity was not entirely dependent on the target antigen, as similar effects were observed with the Ctrl TriTE (Fig. 6a). Due to the non-selective nature of the CD206 TriTE in this model, subsequent efforts were directed towards evaluating the FRβ-targeting BiTEs.

We then assessed the expression of M1-like markers CD64, CD80 and CD86 on CD11b^+^CD64^+^ ascites macrophages remaining after FRβ BiTE treatment. A shift towards a pro-inflammatory macrophage phenotype was observed, with increased expression of CD80 (1.32- and 1.47-fold average increases in gMFI values following FR3 and 3FR BiTE treatment, respectively) and CD86 (1.40- and 1.35-fold average increases in gMFI values following FR3 and 3FR BiTE treatment, respectively) (Fig. 6b). The FRβ-targeting BiTEs activated both CD4^+^ and CD8^+^ endogenous T cell subsets (Fig. 6c), leading to increased IFN-γ levels in the presence and absence of ascites fluid (3FR BiTE, Fig. 6d), or, in the case of the FR3 BiTE, in the absence of ascites fluid only (Fig. 6d). Increased T cell numbers were observed in all ascites samples, with an average fold-increase in CD4^+^ count of 7.9 and 14.5 for the FR3 and 3FR BiTEs, respectively, and an average fold-increase in CD8^+^ count of 8.0 and 13.5 (in the presence of ascites fluid, Fig. 6e). No significant T cell activation or expansion was observed after treatment with the Ctrl BiTEs (Fig. 6c-e).

To further explore the immunological milieu following BiTE treatment, a multiplex immunoassay was performed with conditioned media from two ascites samples (Patients 10 and 15) treated (or not) with the 3FR BiTE or its matched control. Spectrum-wide increases in soluble immunomodulatory factors were observed after 3FR BiTE treatment, with particularly strong fold-increases in IFN-γ, TNF-α, IL-10, IP-10, TARC, IL-4 and IL-1β (average increases above mock-treated samples of 807-, 51.1-, 18.6-, 12.3-, 9.2- and 9.2-fold, respectively) (Additional file 8). Combined, these data suggest a global switch in the cancer microenvironment after FRβ BiTE treatment towards a more inflammatory state.

### An oncolytic group B adenovirus, enadenotucirev, may be utilised for delivery of the TAM-targeting T cell engagers

**Fig. 7:**
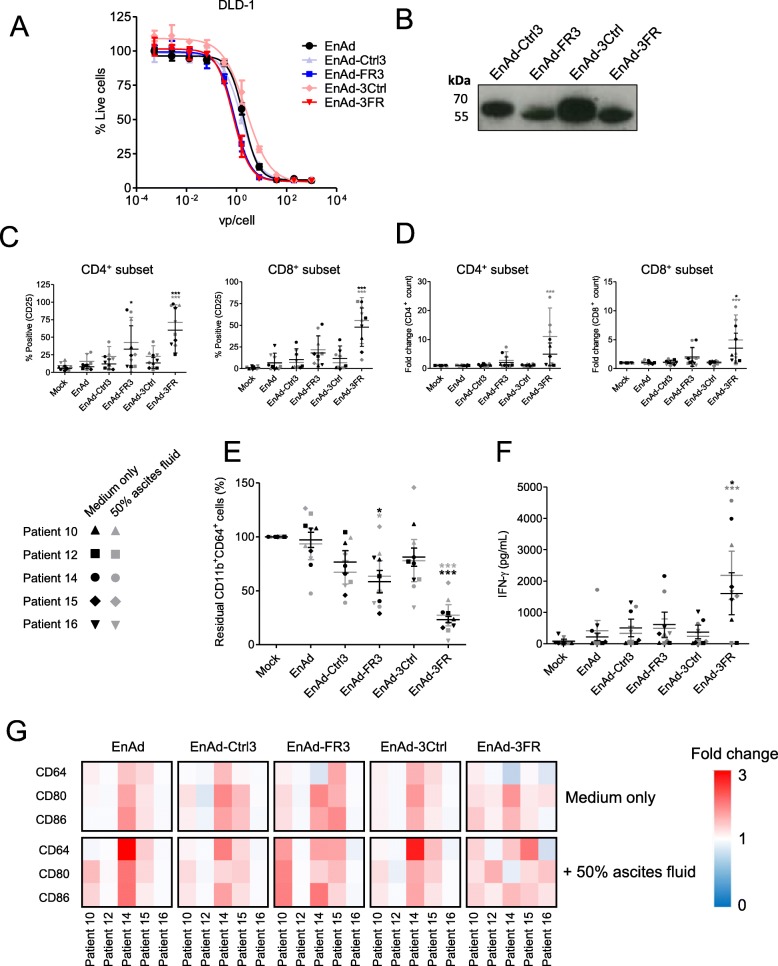
EnAd expressing FRβ-targeting BiTEs activates endogenous ascites T cells to kill ascites macrophages. **a** DLD-1 cells were infected with parental or armed EnAd, and viability assessed four days later by MTT assay. % Live cells was calculated relative to “Mock”-treated cells. **b** DLD-1 cells were infected with armed EnAd at 100 vp/cell. Supernatants were harvested 72 h later and analysed for BiTE expression by western blotting using anti-His primary antibody and an HRP-conjugated anti-mouse secondary antibody. **c**-**g** Total unpurified ascites cells from five patients were infected with 100 vp/cell parental or BiTE-expressing EnAd for five days with or without autologous fluid. **c** Activation of endogenous CD4^+^ and CD8^+^ ascites T cells was assessed by flow cytometric measurement of CD25 expression. **d** CD4^+^ and CD8^+^ cell numbers were determined by adding counting beads to samples immediately prior to antibody staining. Fold-changes in CD4^+^ and CD8^+^ cell count were calculated relative to “Mock”-treated samples. **e**, **g** Cells were stained with anti-CD11b, anti-CD64, anti-CD80 and anti-CD86 antibodies, and a LIVE/DEAD fixable stain, then analysed by flow cytometry. **e** % Live residual CD11b^+^CD64^+^ cells were calculated relative to “Mock”-treated samples. **f** IFN-γ levels in the supernatants were determined by ELISA. **g** Fold-changes in geometric MFI values of CD64, CD80 and CD86 on live CD11b^+^CD64^+^ ascites cells were calculated relative to “Mock”-treated samples for each patient sample. **c**-**f** Data show the grand mean ± SD of five individual patient means (calculated from biological triplicate). **c**-**f** Statistical significance was assessed by two-way ANOVA followed by Bonferroni post-hoc analysis, with each treatment being compared to the relevant “Mock” condition (**c**-**f**) (*, *P* < 0.05; **, *P* < 0.01; ***, *P* < 0.001). (**c**-**g**) (*, *P* < 0.05; **, *P* < 0.01; ***, *P* < 0.001)

EnAd, a chimeric group B oncolytic adenovirus in Phase I/II clinical trials, demonstrates cancer cell-restricted replication and favourable pharmacokinetics following the systemic delivery of three repeated doses [34–36]. EnAd can be engineered to encode biologics that are expressed and secreted by infected tumour cells as the virus replicates, offering a multipronged and tumour-targeted therapeutic strategy [20, 22, 37]. To explore the feasibility of this approach in the context of TAM-targeting T cell engagers, we inserted the FRβ and Ctrl BiTE sequences (in both orientations) downstream of the fibre gene under the control of CMV promoter (Additional file 9). All four BiTE-armed viruses demonstrated comparable oncolytic activity to parental EnAd (Fig. 7a), whilst mediating BiTE secretion into the cell supernatants (Fig. 7b). Indeed, incubation of infected cell supernatants with co-cultures of human lymphocytes and autologous MDMs triggered robust T cell activation and macrophage cytotoxicity (Additional file 10). Supernatants from cells infected with 3FR-BiTE armed EnAd (EnAd-3FR) were particularly effective, achieving significant MDM killing at dilutions as low as 1:1000 (Additional file 10).

We then tested the FRβ BiTE-armed viruses in whole ascites from five cancer patients. In the presence and absence of autologous ascites fluid, EnAd-3FR mediated significant activation and expansion of CD4^+^ and CD8^+^ T cell subsets (Fig. 7c and d). T cell activation by EnAd-FR3 was less pronounced, achieving significance only for the CD4^+^ subset (Fig. 7c). Treatment with EnAd-3FR triggered a robust decline in the number of ascites macrophages, with the average % residual CD11b^+^CD64^+^ cells across the five patient samples reducing to 23.3 and 27.3% in the absence and presence of ascites fluid, respectively (Fig. 7e). EnAd-3FR treatment was additionally associated with a strong increase in IFN-γ production (Fig. 7f). EnAd-FR3-mediated reduction of CD11b^+^CD64^+^ cells was more modest (average % residual CD11b^+^CD64^+^ cells of 58.5 and 63.4% in the absence and presence of ascites fluid, respectively, Fig. 7e), with no significant increases in IFN-γ levels (Fig. 7f).

To assess whether these treatments repolarised the residual CD11b^+^CD64^+^ ascites macrophages, we measured their expression of M1-like markers (Fig. 7g and Additional file 11). For three of five samples tested (Patients 10, 14 and 15), the FRβ BiTE-armed EnAd viruses, as well as parental EnAd and the relevant Ctrl BiTE-armed viruses, triggered a general increase in M1-like marker expression above that of mock-treated cells (Fig. 7g, Additional file 11). For four of five ascites patient samples (Patients 10, 12, 15 and 16), the FRβ BiTE-armed viruses (in one or both BiTE orientations) achieved higher fold-increases in the expression of one or more M1 marker(s) than parental EnAd or Ctrl BiTE-armed viruses (Fig. 7g, Additional file 11). Together, these data demonstrate that FRβ BiTE-armed EnAd viruses can trigger activation and expansion of T cells in malignant ascites, leading to depletion of endogenous macrophages and up-regulation of pro-inflammatory macrophage markers.

## Discussion

Here, we have developed a powerful new therapeutic strategy for targeting TAMs. By engineering T cell engagers to recognise M2-like macrophage markers, we have enabled depletion of cancer-promoting TAM subsets, whilst leaving those with anti-tumour potential unharmed. Expression of these potent biologics must be restricted to within the tumour microenvironment, for example with engineered oncolytic viruses. As a proof-of-concept, we encoded our T cell engagers within EnAd, demonstrating robust BiTE expression without compromising oncolytic activity.

Both free and virally-delivered TAM-targeting T cell engagers activated endogenous T cells in malignant ascites, despite the immune-suppressive nature of these samples (Figs. 6 and 7). Activity was most impressive with the FRβ-targeting BiTEs, which triggered robust T cell activation and expansion, IFN-γ production and depletion of ascites macrophages. Importantly, surviving macrophages exhibited a trend towards increased M1-like macrophage marker expression, suggesting either: i) selective targeting of TAMs with the highest expression levels of FRβ, sparing those with more “M1-like” phenotypes, and/or ii) incomplete macrophage cytotoxicity, with repolarisation of remaining cells due to BiTE-induced pro-inflammatory signals.

Our approach offers several opportunities for synergy. Clinical experience has revealed increased T cell infiltration of tumours following treatment with OVs including EnAd [34, 38–42]. Increased intratumoural T cells may enhance BiTE efficacy by increasing the E:T ratio. On the other hand, BiTEs may redirect anti-viral T cells from virus-infected cells, facilitating greater viral spread [43]. The impact of TAM removal on OV therapy is difficult to predict [44]; however, several studies suggest improved OV efficacy following TAM depletion. In glioma-bearing mice, cyclophosphamide enhanced oncolytic adenovirus replication and prolonged virus-mediated transgene expression [45]. Moreover, macrophage depletion (with clodronate or trabectidin) improved the anti-tumour efficacy of an oncolytic herpes simplex virus in Ewing’s sarcoma xenograft models, in this case due to a shift in the tumour microenvironment towards a more pro-inflammatory state [46].

To the best of our knowledge, we are the first to engineer a single-chain T cell engager with bivalent CD3 binding. Bivalent CD3 binding increased the efficacy of the parental T cell engager, markedly improving its activity in immunosuppressive conditions (Fig. 5). Nevertheless, the therapeutic window for the bivalent CD3-binding T cell engager appeared reduced, with non-specific T cell activation and cytotoxicity induced by its matched control at higher doses, warranting caution in the use of such constructs. Another interesting finding was that CD28-containing TriTEs trigger non-specific T cell activation, with no apparent requirement for a target antigen (Fig. 4).

Despite similar performances in healthy PBMC models (Fig. 2), the CD206 BiTE was inferior to the FRβ BiTE in more clinically-relevant settings (Figs. 3 and 6). Several factors may underlie this finding. T cell activation requires CD45 exclusion from the immunological synapse, which occurs when the two membranes are brought into close apposition [47]. The presence of a bulky antigen in the synapse may therefore decrease T cell activation. Indeed, smaller antigens were found to facilitate superior BiTE-mediated T cell activation [48]. At 170–180 kDa, CD206 may represent a more challenging target for T cell-based therapies than FRβ (30–40 kDa). Another explanation relates to target antigen density, which is known to influence BiTE efficacy [49]. In healthy MDM models, we observed higher levels of CD206 than FRβ (Additional file 2). By contrast, levels of FRβ on ascites macrophages were greater than that of CD206 (Fig. 1). Ascites macrophages most likely derive from peritoneal (i.e. tissue-resident) macrophages, as opposed to infiltrating monocytes [50]. The differing levels of CD206 and FRβ observed on the macrophages in our study may reflect their different ontogenies. The origins of TAMs in solid tumours is a subject of debate, likely depending on the tumour type/stage. A third factor possibly limiting CD206 BiTE efficacy may be the presence of soluble CD206 in ascites fluid (Fig. 3d). However, we found no significant correlation between soluble CD206 levels and T cell activation by the BiTE (Additional file 12), suggesting a role of additional factors.

An important consequence of this treatment strategy may be the activation and expansion of tumour-infiltrating lymphocytes (TILs). Increasingly, it appears that TILs can recognise tumour-associated antigens [51–53]. This raises the possibility that BiTE-activated/expanded TILs will proceed to mediate cytotoxic activity via their own human leukocyte antigen-restricted specificity if concentrations of the BiTE fall sufficiently, perhaps diversifying the anti-cancer effect.

## Conclusions

Here, we have generated novel T cell engagers capable of redirecting endogenous T cell cytotoxicity towards M2-like TAMs, whilst leaving those with anti-tumour potential unharmed. We have engineered an oncolytic adenovirus, EnAd, to express the TAM-targeting T cell engagers without compromising its oncolytic activity, yielding a multi-pronged therapeutic modality to simultaneously target cancer cells and immunosuppressive TAMs. Altogether, we foresee that removal of cancer-promoting TAMs, combined with the immune-stimulatory effects of BiTEs and OVs, will provide a powerful therapeutic approach for removing barriers to anti-tumour immunity in patients with cancer.

## Supplementary information

**Additional file 1.** Dot blot analysis of BiTE/TriTEs. A-C, Two-fold serial dilutions of BiTE/TriTE-containing supernatants from transfected HEK293A cells were concentrated and applied to a nitrocellulose membrane, alongside a 10xHis-tagged protein of known concentration. Membranes were probed with anti-His primary antibody and HRP-conjugated anti-mouse secondary antibody. A, An exemplary dot blot of a 10xHis-tagged protein of known concentration. B, An exemplary standard curve to determine BiTE/TriTE concentration, as generated by measuring dot intensity with ImageJ software. C, Dot blot analyses BiTE/TriTE-containing supernatants.

**Additional file 2.** CD206 and FRβ expression by MDMs. MDMs from healthy donor PBMCs were polarised as indicated and stained with PE-conjugated anti-CD206 or anti-FRβ antibodies, or isotype controls, then analysed by flow cytometry. Representative histograms are displayed.

**Additional file 3. **CD206- and FRβ-targeting BiTEs activate primary human T cells in the presence of autologous target MDMs. A-C, T cells were co-cultured with polarised autologous MDMs, and activation assessed by flow cytometric measurement of CD69 and CD107a (24 h after BiTE addition), and HLA-DR (96 h after BiTE addition). Data show mean ± SD of Statistical analysis was performed by two-way ANOVA with Bonferroni post-hoc tests comparing with the relevant “Mock” condition (*, *P* < 0.05; **, *P* < 0.01; ***, *P* < 0.001).

**Additional file 4. **CD206- and FRβ BiTE-induced T cell-mediated killing of MDMs is dependent on the perforin pathway. T cells were co-cultured with autologous CFSE-stained MDMs and treated (or not) with the indicated BiTEs for 96 h, at which point % Live cells were calculated with Celigo image cytometry. For inhibition of perforin, T cells were pre-treated for 2 h with Concanamycin A (100 ng/mL), or an equivalent concentration of vehicle control (DMSO), then washed prior to BiTE addition. Inhibitors of Fas/FasL (anti-Fas antibody, clone ZB4, 2 μg/mL) and TRAIL (*TRAIL*-*R1-Fc, 1* μg/mL) were added at the point of BiTE treatment and not removed. Data show mean ± SD of biological triplicates. Statistical significance was assessed by two-way ANOVA followed by Bonferroni post-hoc analysis, with each treatment being compared to the relevant “Mock” condition (*, *P* < 0.05; **, *P* < 0.01; ***, *P* < 0.001).

**Additional file 5. **A CD206-targeting TriTE outperforms the parental BiTE at an E:T ratio of 2:1. MDMs were polarised, CFSE-stained, and co-cultured for 96 h with T cells at a fixed E:T ratio of 2:1, in the presence of increasing BiTE/TriTE concentrations. % Live cells were calculated with propidium iodide staining and Celigo image cytometry. Data show mean ± SD of biological triplicates. Statistical significance was assessed by two-way ANOVA followed by Bonferroni post-hoc analysis, with each treatment being compared to the relevant “Mock” condition (*, *P* < 0.05; **, *P* < 0.01; ***, *P* < 0.001).

**Additional file 6. **Characterisation of a FRβ BiTE with reversed scFv domains. A, Schematic representations of the 3FR BiTE and its matched control. B, Western blotting analysis of supernatants from HEK293A cells 48 h after transfection with 3FR and 3Ctrl BiTE expression plasmids. Blots were probed with a mouse anti-His primary antibody, then HRP-conjugated anti-mouse secondary antibody. C, Human MDMs were polarised, stained with CFSE, and treated with T cells (10:1 E:T ratio) and increasing concentrations of BiTEs. Macrophage cytotoxicity was assessed 96 h later by propidium iodide staining and Celigo image cytometry. D, Monocyte-derived macrophages were CFSE-stained and treated with the indicated concentrations of BiTE in the presence or absence of T cells (10:1 E:T ratio). 96 h later, cytotoxicity was assessed by propidium iodide staining and analysis with a Celigo image cytometer. E, T cell activation in the presence or absence of target cells was assessed by flow cytometric measurement of CD25 expression 96 h after BiTE addition. Data show mean ± SD of biological triplicates (C, D and E). Statistical analysis was performed by two-way ANOVA with Bonferroni post-hoc tests comparing with the relevant “Mock” condition (D and E) (*, *P* < 0.05; **, *P* < 0.01; ***, *P* < 0.001).

**Additional file 7.** Representative flow cytometric dot plots of CD206 and FRβ expression by whole ascites cells from five cancer patients.

**Additional file 8.** 3FR BiTE treatment triggers wide-ranging increases in immunomodulatory cytokines and chemokines. Cell-free supernatants from two ascites samples treated (or not) for five days with 3Ctrl or 3FR BiTE were assessed using a 13-plex immunoassay. Fold-increases in cytokine/chemokine levels were calculated relative to “Mock”-treated samples. Data show mean ± SD of biological triplicates.

**Additional file 9.** A schematic representation of the EnAd genome encoding a BiTE transgene under the control of the CMV promoter.

**Additional file 10. **Supernatants from cells infected with BiTE-armed EnAd trigger T cell-mediated cytotoxicity of macrophages. A,B, DLD-1 cells were infected with the indicated viruses at a dose of 100 virus particles/cell. 72 h later, supernatants were harvested and applied at the indicated dilutions to co-cultures of PBMC-derived T cells and CFSE-stained monocyte-derived macrophages (MDM). A, After four days’ co-culture, MDM killing was assessed with propidium iodide staining and Celigo image cytometry. B, T cell activation was assessed by measuring CD25 expression with flow cytometry. Data show mean ± SD of biological triplicates (A,B). Statistical analysis was performed by two-way ANOVA with Bonferroni post-hoc tests comparing with the relevant “Mock” condition (A), or with one-way ANOVA followed by Dunnett’s post-hoc analysis compared with “Mock”-treated cells (B). (*, *P* < 0.05; **, *P* < 0.01; ***, *P* < 0.001).

**Additional file 11. **Expression of M1 macrophage markers on ascites from individual patient samples following virus treatment. Total unpurified ascites cells from different patients were infected with 100 vp/cell parental or BiTE-expressing EnAd for 5 days in the presence or absence of autologous fluid. Cells were stained with anti-CD11b, anti-CD64, anti-CD80 and anti-CD86 antibodies, as well as a LIVE/DEAD fixable stain, then analysed by flow cytometry. Fold-changes in geometric mean fluorescence intensity (MFI) values of CD64, CD80 and CD86 on live CD11b^+^CD64^+^ ascites cells were calculated relative to “Mock”-treated samples. Data show mean ± SD of biological triplicates. Statistical significance was assessed by two-way ANOVA followed by Bonferroni post-hoc analysis, with each treatment being compared to either “EnAd” alone or, in the case of EnAd, “Mock”. (*, *P* < 0.05; **, *P* < 0.01; ***, *P* < 0.001).

**Additional file 12.** Relationship between soluble CD206 levels in ascites fluid and T cell activation by the CD206 BiTE. A,B, Monocyte-derived macrophages were co-cultured with T cells and 50 nM CD206 BiTE, in the presence or absence of the indicated patient ascites fluids. T cell activation, as assessed by CD25 expression (geometric MFI values in A, % positive in B), was determined 72 h later. Levels of soluble CD206 in the supernatant were determined by ELISA. Pearson correlation calculations were performed with Graphpad Prism software. Data show mean ± SD of biological triplicates.

**Additional file 13.** Estimates of virus particles (vp)/mL for purified virus stocks, as quantified by Picogreen and HPLC.

**Additional file 14.** Cellular composition of malignant ascites samples used in Figs. 6 and 7.

**Additional file 15.** Patient indications for malignant ascites samples used in this study.

## Data Availability

Not applicable.

## Abbreviations

BiTE: bi-valent T cell engager
EnAd: enadenotucirev
FRβ: Folate receptor β
MDM: Monocyte-derived macrophage
OV: Oncolytic virus
PBMC: Peripheral blood mononuclear cells
TAM: Tumour-associated macrophage
TriTE: tri-valent T cell engager
