# Supplementary figures and images for: Bi- and tri-valent T cell engagers deplete tumour-associated macrophages in cancer patient samples
Source: J Immunother Cancer. 2019 Nov 21;7:320. doi: 10.1186/s40425-019-0807-6 (PMC6873687; doi:10.1186/s40425-019-0807-6)

# Additional File 1

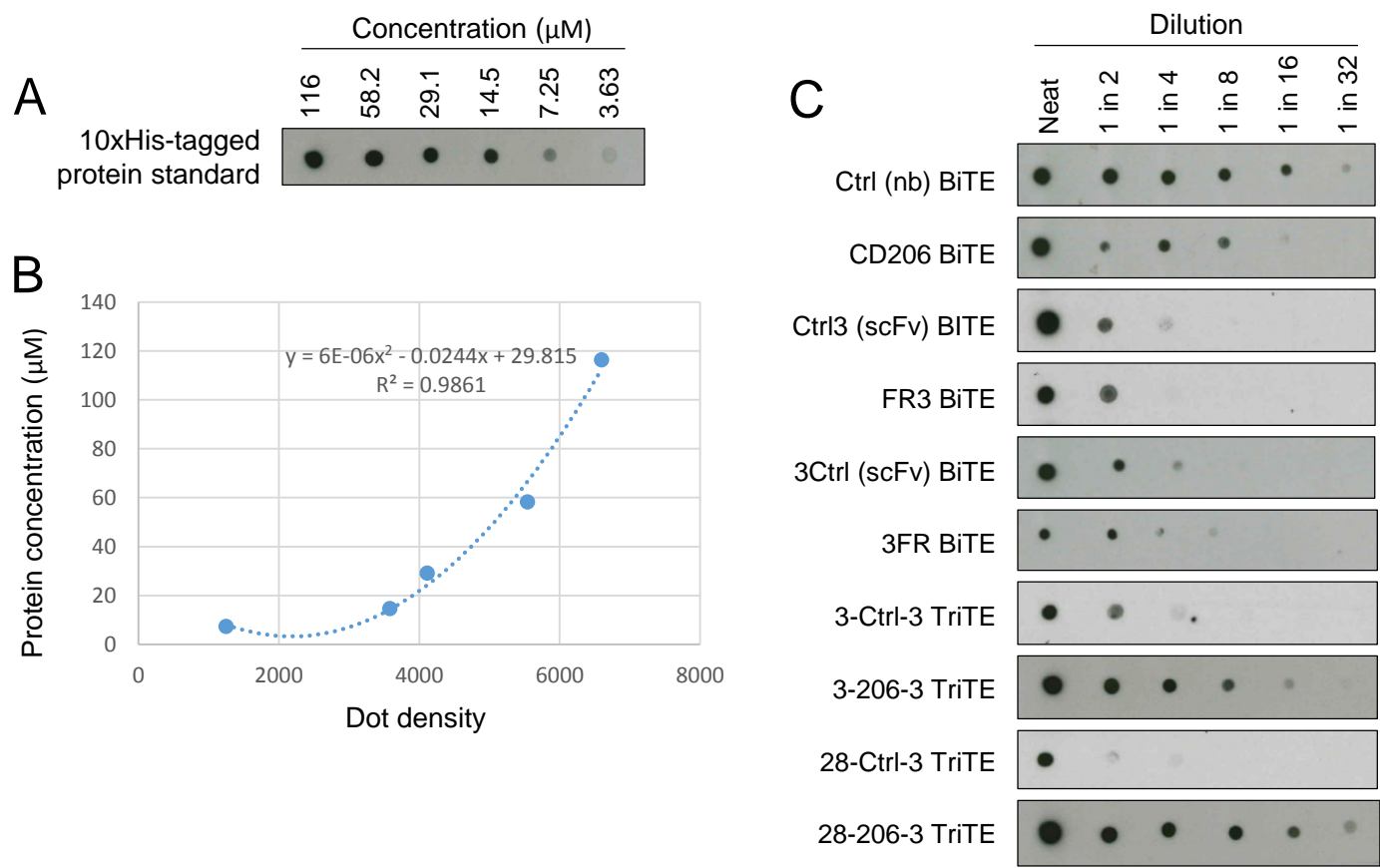

Supplement: Supplementary file 1 — Additional file 1. Dot blot analysis of BiTE/TriTEs. A-C, Two-fold serial dilutions of BiTE/TriTE-containing supernatants from transfected HEK293A cells were concentrated and applied to a nitrocellulose membrane, alongside a 10xHis-tagged protein of known concentration. Membranes were probed with anti-His primary antibody and HRP-conjugated anti-mouse secondary antibody. A, An exemplary dot blot of a 10xHis-tagged protein of known concentration. B, An exemplary standard curve to determine BiTE/TriTE concentration, as generated by measuring dot intensity with ImageJ software. C, Dot blot analyses BiTE/TriTE-containing supernatants. [file 40425_2019_807_MOESM1_ESM.pdf]

# Additional File 2

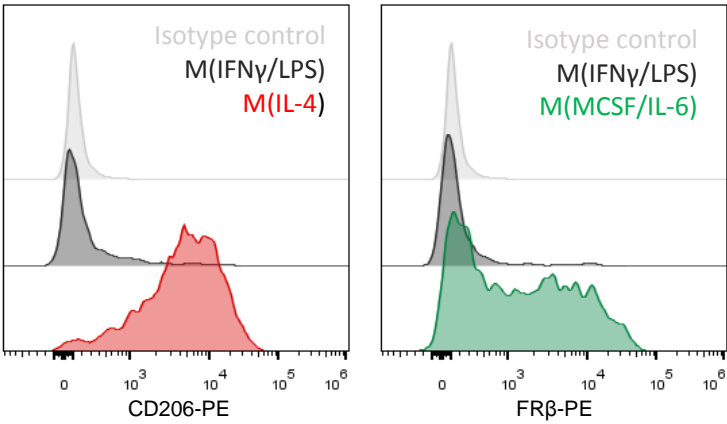

Supplement: Supplementary file 2 — Additional file 2. CD206 and FRβ expression by MDMs. MDMs from healthy donor PBMCs were polarised as indicated and stained with PE-conjugated anti-CD206 or anti-FRβ antibodies, or isotype controls, then analysed by flow cytometry. Representative histograms are displayed. [file 40425_2019_807_MOESM2_ESM.pdf]

# Additional File 3

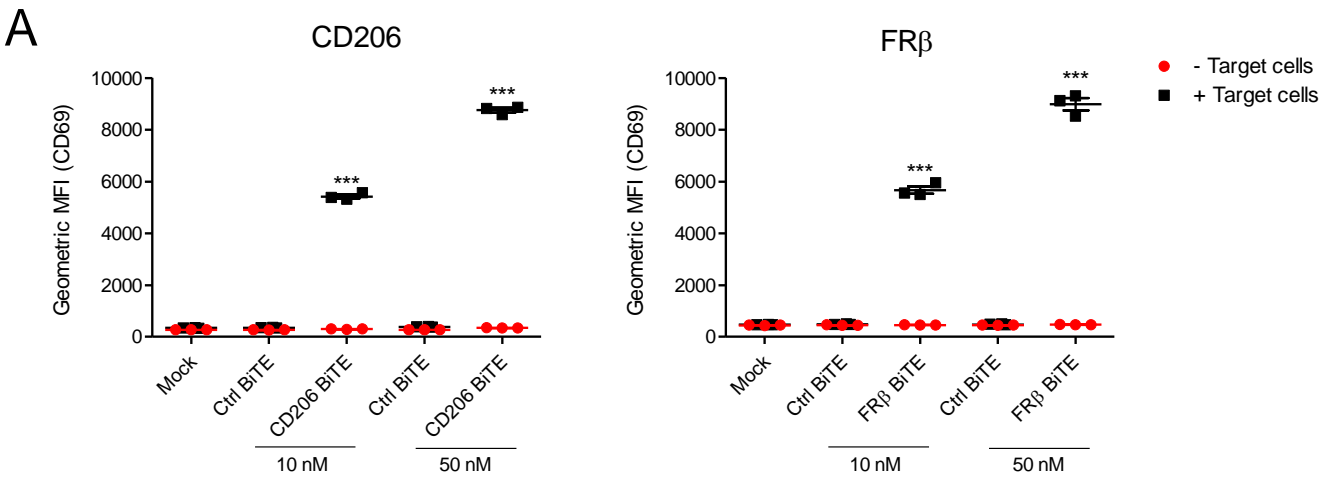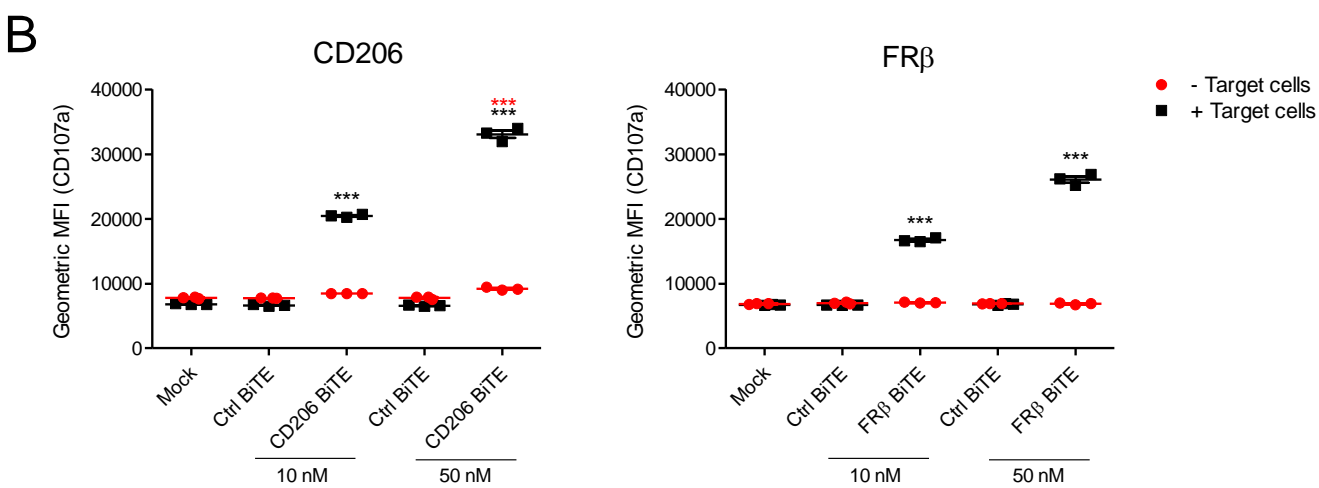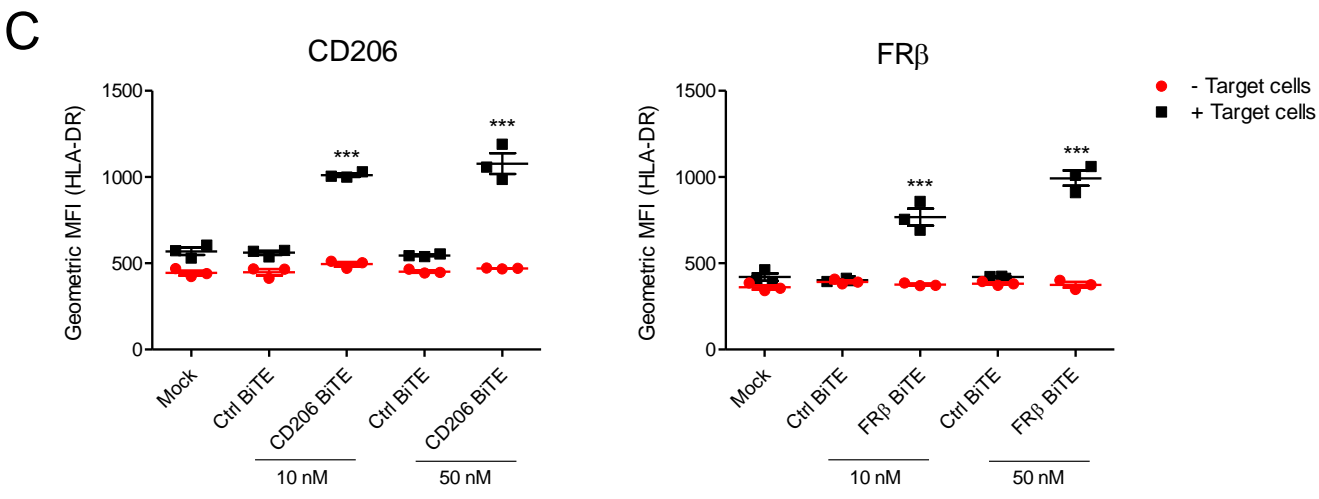

Supplement: Supplementary file 3 — Additional file 3. CD206- and FRβ-targeting BiTEs activate primary human T cells in the presence of autologous target MDMs. A-C, T cells were co-cultured with polarised autologous MDMs, and activation assessed by flow cytometric measurement of CD69 and CD107a (24 h after BiTE addition), and HLA-DR (96 h after BiTE addition). Data show mean ± SD of Statistical analysis was performed by two-way ANOVA with Bonferroni post-hoc tests comparing with the relevant “Mock” condition (*, P < 0.05; **, P < 0.01; ***, P < 0.001). [file 40425_2019_807_MOESM3_ESM.pdf]

# Additional File 4

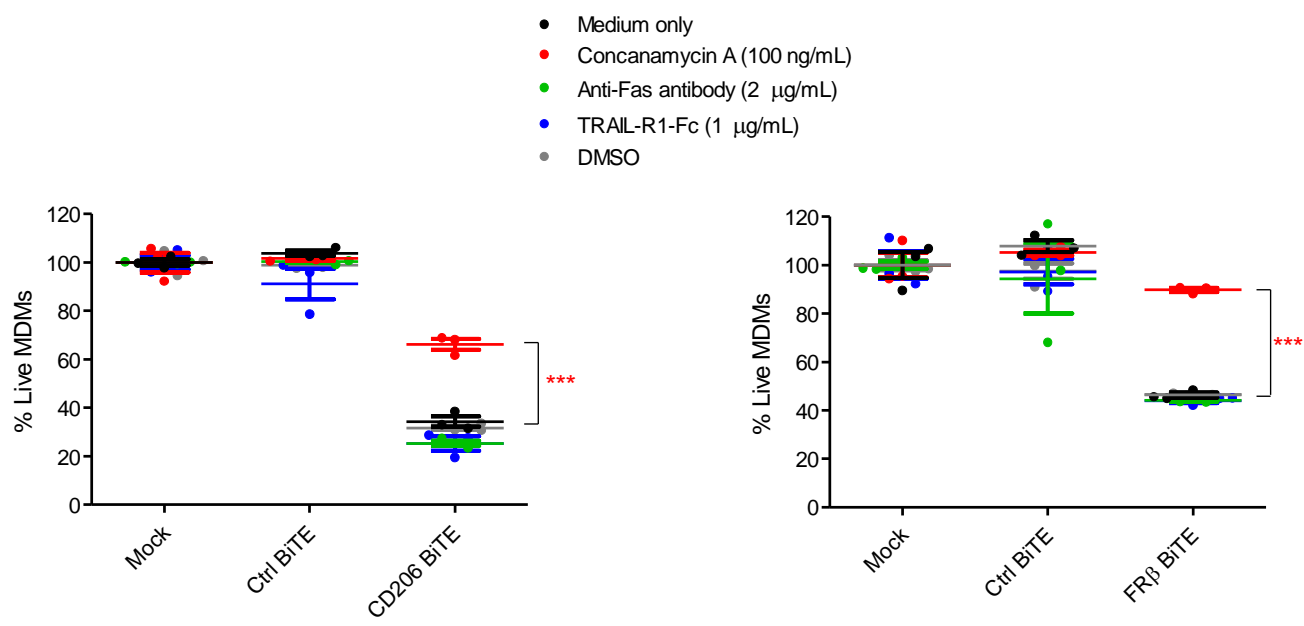

Supplement: Supplementary file 4 — Additional file 4. CD206- and FRβ BiTE-induced T cell-mediated killing of MDMs is dependent on the perforin pathway. T cells were co-cultured with autologous CFSE-stained MDMs and treated (or not) with the indicated BiTEs for 96 h, at which point % Live cells were calculated with Celigo image cytometry. For inhibition of perforin, T cells were pre-treated for 2 h with Concanamycin A (100 ng/mL), or an equivalent concentration of vehicle control (DMSO), then washed prior to BiTE addition. Inhibitors of Fas/FasL (anti-Fas antibody, clone ZB4, 2 μg/mL) and TRAIL (TRAIL-R1-Fc, 1 μg/mL) were added at the point of BiTE treatment and not removed. Data show mean ± SD of biological triplicates. Statistical significance was assessed by two-way ANOVA followed by Bonferroni post-hoc analysis, with each treatment being compared to the relevant “Mock” condition (*, P < 0.05; **, P < 0.01; ***, P < 0.001). [file 40425_2019_807_MOESM4_ESM.pdf]

# Additional File 5

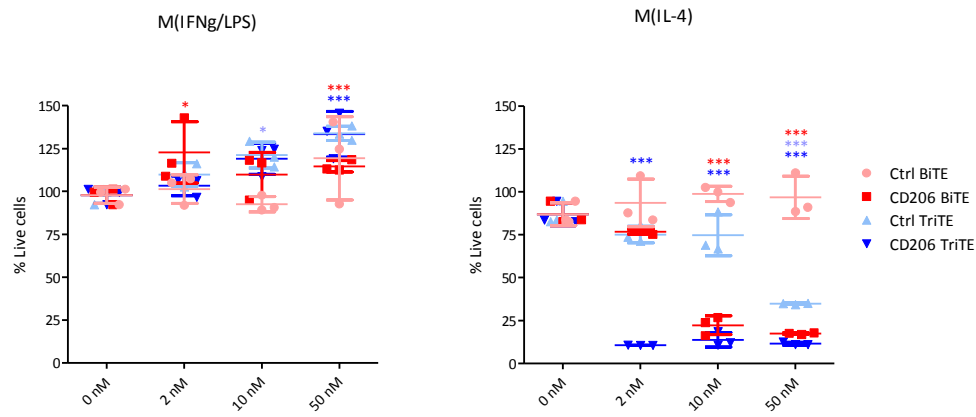

Supplement: Supplementary file 5 — Additional file 5. A CD206-targeting TriTE outperforms the parental BiTE at an E:T ratio of 2:1. MDMs were polarised, CFSE-stained, and co-cultured for 96 h with T cells at a fixed E:T ratio of 2:1, in the presence of increasing BiTE/TriTE concentrations. % Live cells were calculated with propidium iodide staining and Celigo image cytometry. Data show mean ± SD of biological triplicates. Statistical significance was assessed by two-way ANOVA followed by Bonferroni post-hoc analysis, with each treatment being compared to the relevant “Mock” condition (*, P < 0.05; **, P < 0.01; ***, P < 0.001). [file 40425_2019_807_MOESM5_ESM.pdf]

# Additional File 6

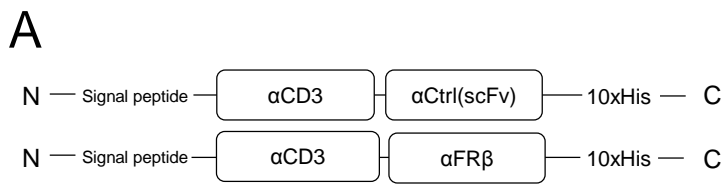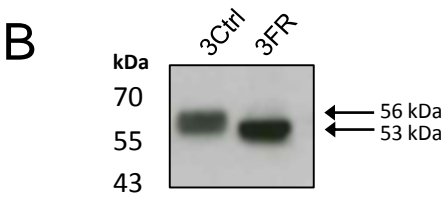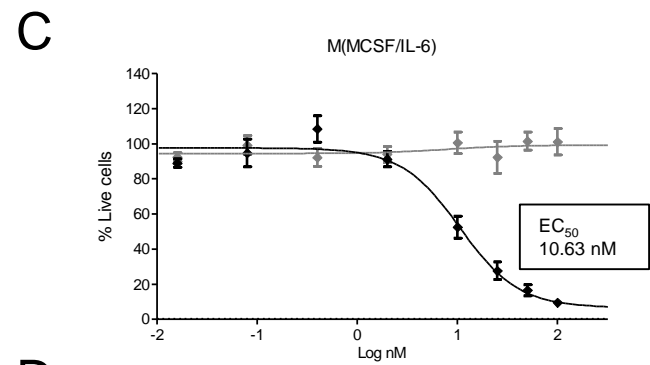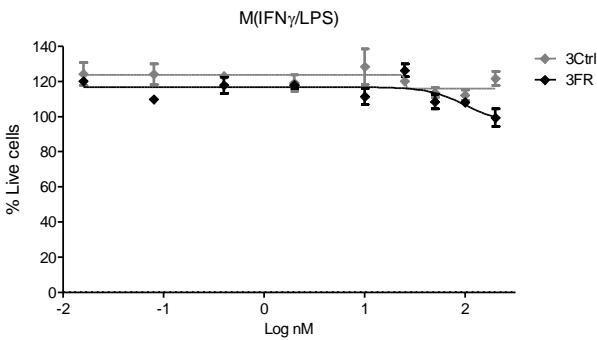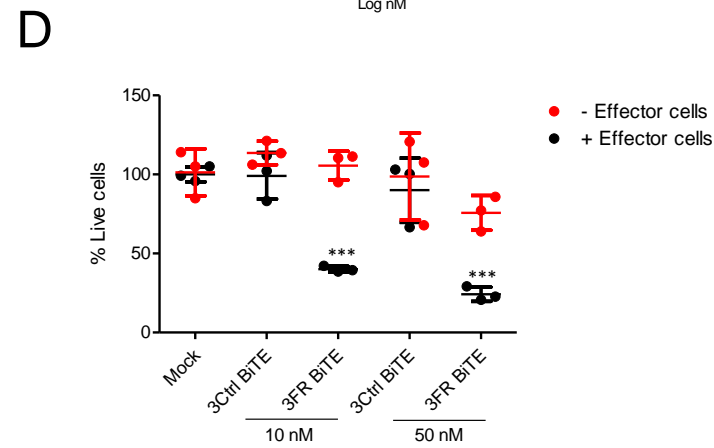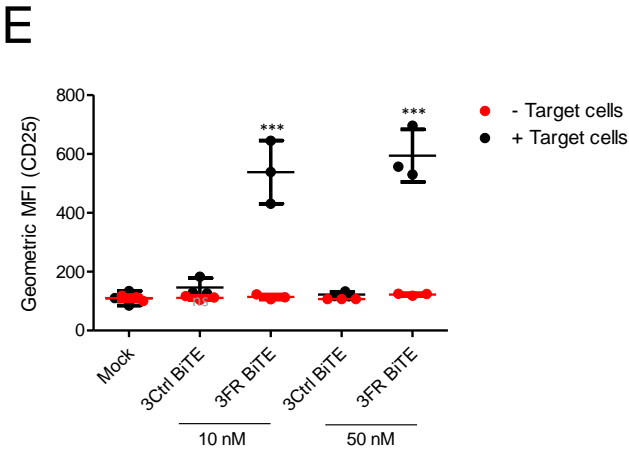

Supplement: Supplementary file 6 — Additional file 6. Characterisation of a FRβ BiTE with reversed scFv domains. A, Schematic representations of the 3FR BiTE and its matched control. B, Western blotting analysis of supernatants from HEK293A cells 48 h after transfection with 3FR and 3Ctrl BiTE expression plasmids. Blots were probed with a mouse anti-His primary antibody, then HRP-conjugated anti-mouse secondary antibody. C, Human MDMs were polarised, stained with CFSE, and treated with T cells (10:1 E:T ratio) and increasing concentrations of BiTEs. Macrophage cytotoxicity was assessed 96 h later by propidium iodide staining and Celigo image cytometry. D, Monocyte-derived macrophages were CFSE-stained and treated with the indicated concentrations of BiTE in the presence or absence of T cells (10:1 E:T ratio). 96 h later, cytotoxicity was assessed by propidium iodide staining and analysis with a Celigo image cytometer. E, T cell activation in the presence or absence of target cells was assessed by flow cytometric measurement of CD25 expression 96 h after BiTE addition. Data show mean ± SD of biological triplicates (C, D and E). Statistical analysis was performed by two-way ANOVA with Bonferroni post-hoc tests comparing with the relevant “Mock” condition (D and E) (*, P < 0.05; **, P < 0.01; ***, P < 0.001). [file 40425_2019_807_MOESM6_ESM.pdf]

# Additional File 7

Patient 10

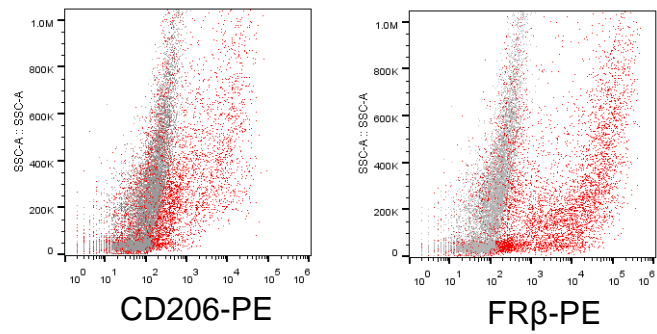

Patient 12

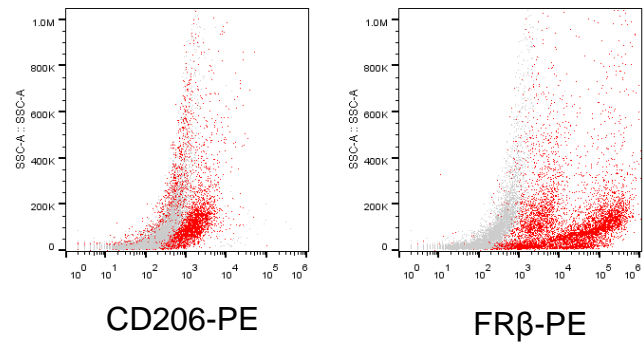

Patient 14

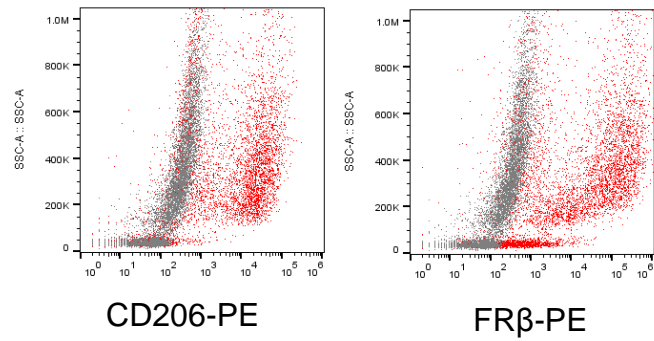

Patient 15

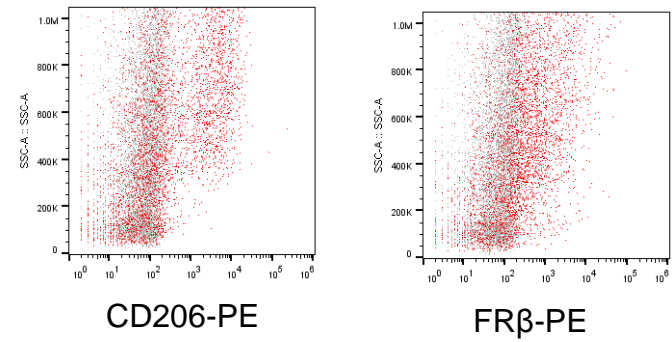

Patient 16

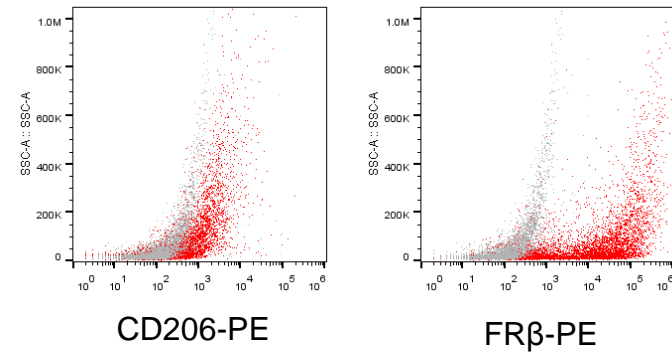

Supplement: Supplementary file 7 — Additional file 7. Representative flow cytometric dot plots of CD206 and FRβ expression by whole ascites cells from five cancer patients. [file 40425_2019_807_MOESM7_ESM.pdf]

# Additional File 8

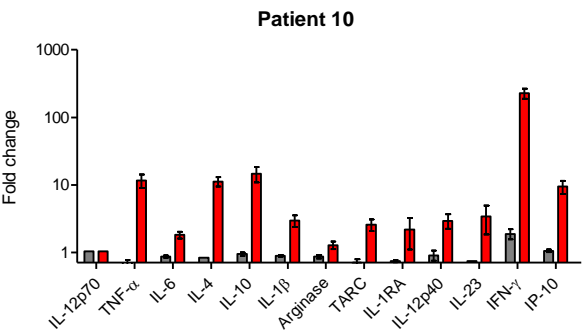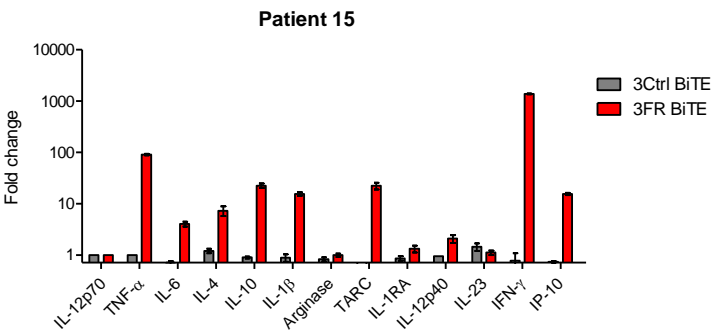

Supplement: Supplementary file 8 — Additional file 8. 3FR BiTE treatment triggers wide-ranging increases in immunomodulatory cytokines and chemokines. Cell-free supernatants from two ascites samples treated (or not) for five days with 3Ctrl or 3FR BiTE were assessed using a 13-plex immunoassay. Fold-increases in cytokine/chemokine levels were calculated relative to “Mock”-treated samples. Data show mean ± SD of biological triplicates. [file 40425_2019_807_MOESM8_ESM.pdf]

Additional File 9

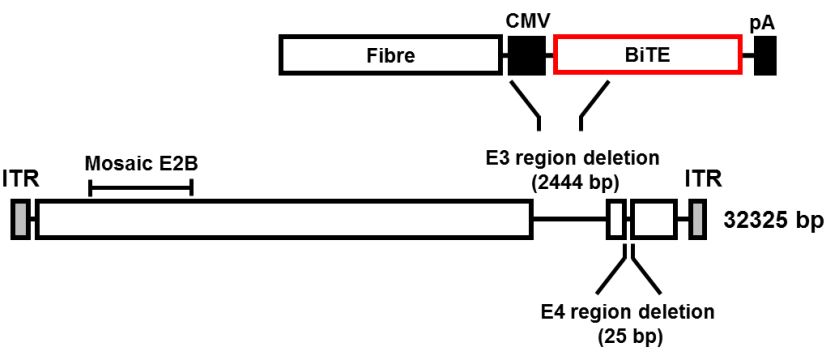

Supplement: Supplementary file 9 — Additional file 9. A schematic representation of the EnAd genome encoding a BiTE transgene under the control of the CMV promoter. [file 40425_2019_807_MOESM9_ESM.pdf]

A

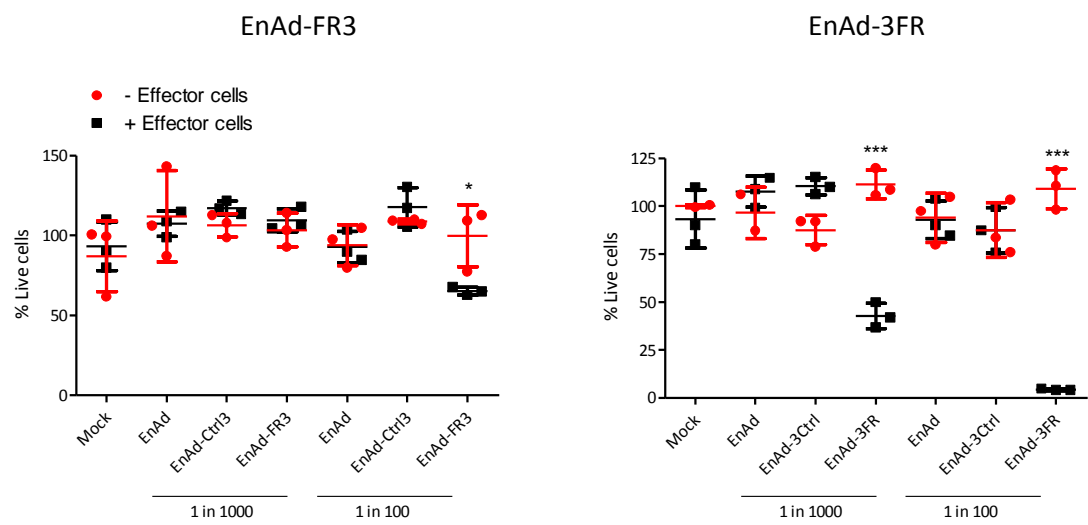

B

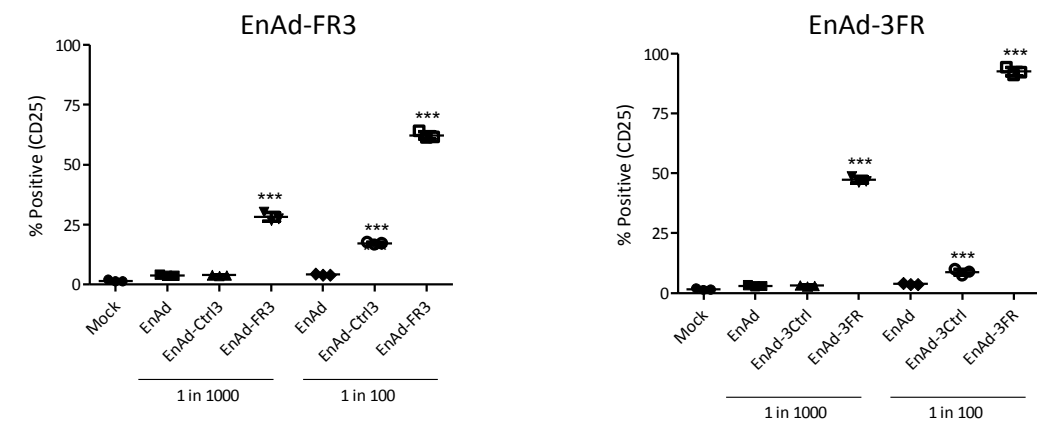

Supplement: Supplementary file 10 — Additional file 10. Supernatants from cells infected with BiTE-armed EnAd trigger T cell-mediated cytotoxicity of macrophages. A,B, DLD-1 cells were infected with the indicated viruses at a dose of 100 virus particles/cell. 72 h later, supernatants were harvested and applied at the indicated dilutions to co-cultures of PBMC-derived T cells and CFSE-stained monocyte-derived macrophages (MDM). A, After four days’ co-culture, MDM killing was assessed with propidium iodide staining and Celigo image cytometry. B, T cell activation was assessed by measuring CD25 expression with flow cytometry. Data show mean ± SD of biological triplicates (A,B). Statistical analysis was performed by two-way ANOVA with Bonferroni post-hoc tests comparing with the relevant “Mock” condition (A), or with one-way ANOVA followed by Dunnett’s post-hoc analysis compared with “Mock”-treated cells (B). (*, P < 0.05; **, P < 0.01; ***, P < 0.001). [file 40425_2019_807_MOESM10_ESM.pdf]

# Additional File 11

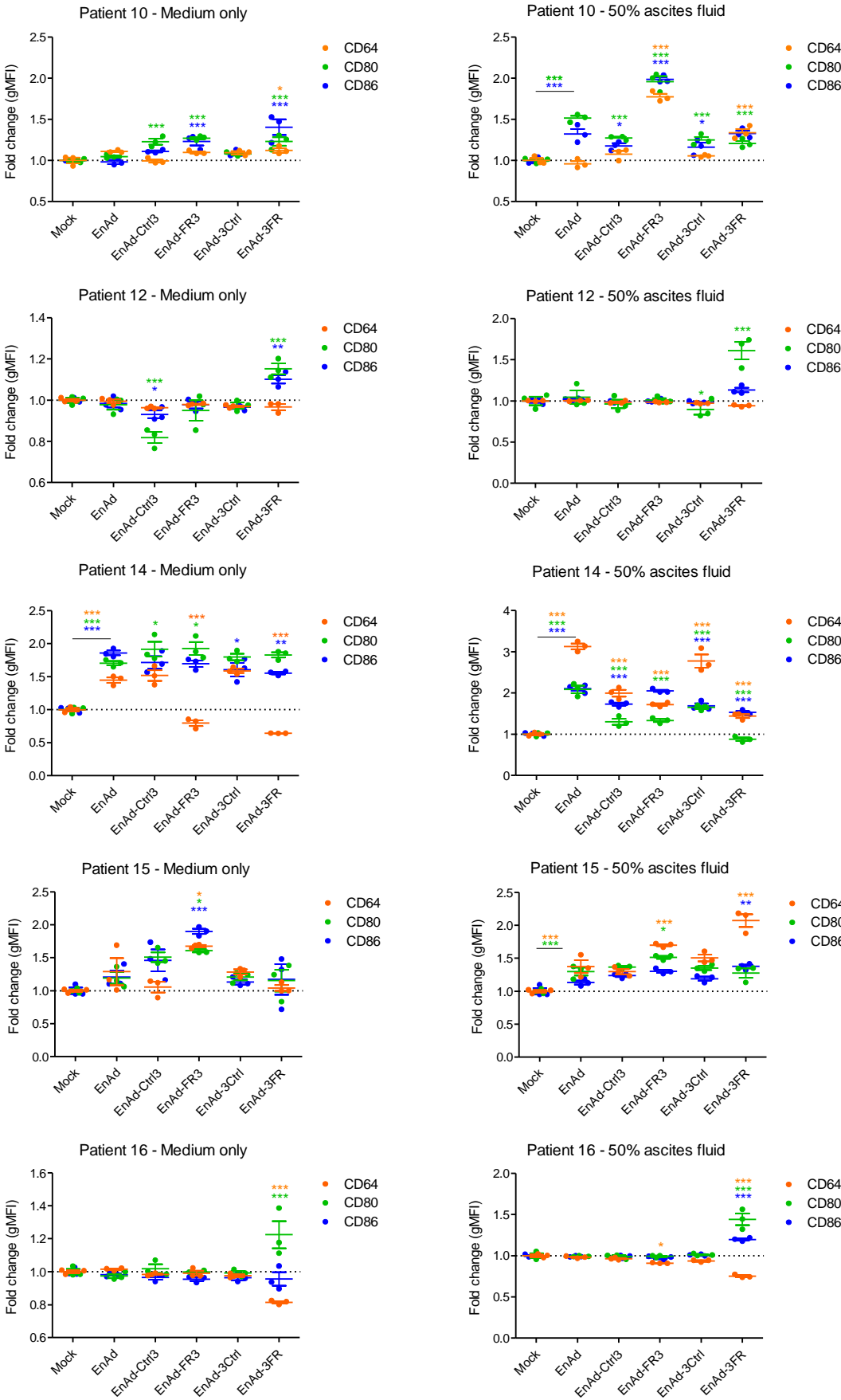

Supplement: Supplementary file 11 — Additional file 11. Expression of M1 macrophage markers on ascites from individual patient samples following virus treatment. Total unpurified ascites cells from different patients were infected with 100 vp/cell parental or BiTE-expressing EnAd for 5 days in the presence or absence of autologous fluid. Cells were stained with anti-CD11b, anti-CD64, anti-CD80 and anti-CD86 antibodies, as well as a LIVE/DEAD fixable stain, then analysed by flow cytometry. Fold-changes in geometric mean fluorescence intensity (MFI) values of CD64, CD80 and CD86 on live CD11b+CD64+ ascites cells were calculated relative to “Mock”-treated samples. Data show mean ± SD of biological triplicates. Statistical significance was assessed by two-way ANOVA followed by Bonferroni post-hoc analysis, with each treatment being compared to either “EnAd” alone or, in the case of EnAd, “Mock”. (*, P < 0.05; **, P < 0.01; ***, P < 0.001). [file 40425_2019_807_MOESM11_ESM.pdf]

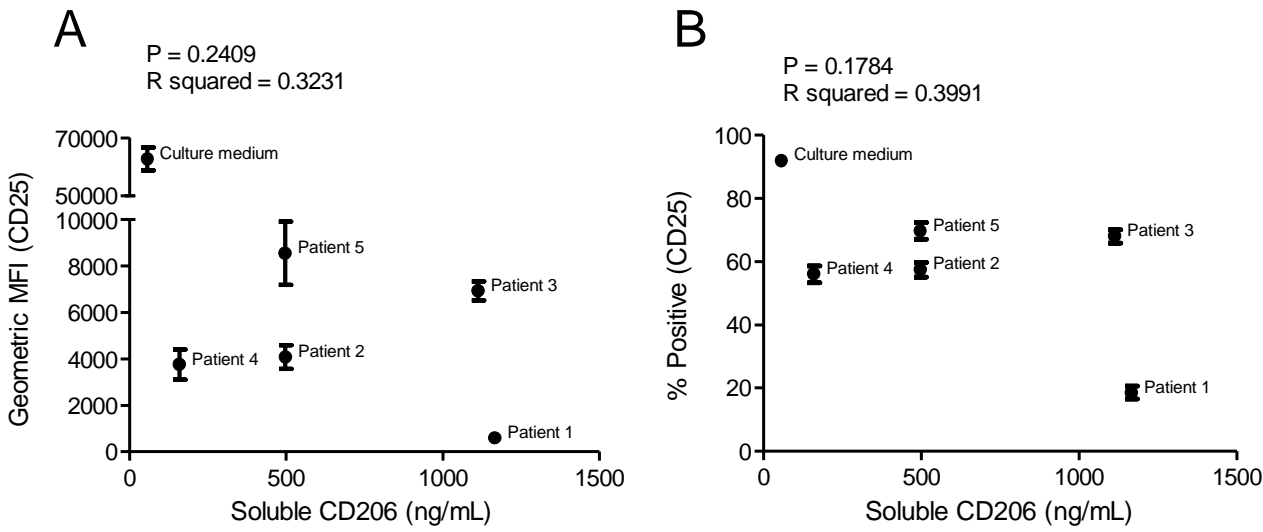

Supplement: Supplementary file 12 — Additional file 12. Relationship between soluble CD206 levels in ascites fluid and T cell activation by the CD206 BiTE. A,B, Monocyte-derived macrophages were co-cultured with T cells and 50 nM CD206 BiTE, in the presence or absence of the indicated patient ascites fluids. T cell activation, as assessed by CD25 expression (geometric MFI values in A, % positive in B), was determined 72 h later. Levels of soluble CD206 in the supernatant were determined by ELISA. Pearson correlation calculations were performed with Graphpad Prism software. Data show mean ± SD of biological triplicates. [file 40425_2019_807_MOESM12_ESM.pdf]
