# Supplementary material for: Bi- and tri-valent T cell engagers deplete tumour-associated macrophages in cancer patient samples
Source: J Immunother Cancer. 2019 Nov 21;7:320. doi: 10.1186/s40425-019-0807-6 (PMC6873687; doi:10.1186/s40425-019-0807-6)
Supplement: Supplementary file 13 — Additional file 13. Estimates of virus particles (vp)/mL for purified virus stocks, as quantified by Picogreen and HPLC. [file 40425_2019_807_MOESM13_ESM.pdf]

Additional File 13

Estimates of virus particles (vp)/mL for purified virus stocks, as quantified by Picogreen and HPLC

	**Picogreen**	**HPLC**
**EnAd**	2.73×10^{11}	3.83×10^{11}
**EnAd-Ctrl3**	5.41865×10^{11}	8.02×10^{11}
**EnAd-FR3**	3.27462×10^{11}	5.51×10^{11}
**EnAd-3Ctrl**	1.58983×10^{11}	2.40×10^{11}
**EnAd-3FR**	9.24542×10^{11}	1.75×10^{12}
