# Supplementary material for: Bi- and tri-valent T cell engagers deplete tumour-associated macrophages in cancer patient samples
Source: J Immunother Cancer. 2019 Nov 21;7:320. doi: 10.1186/s40425-019-0807-6 (PMC6873687; doi:10.1186/s40425-019-0807-6)
Supplement: Supplementary file 14 — Additional file 14. Cellular composition of malignant ascites samples used in Figs. 6 and 7. [file 40425_2019_807_MOESM14_ESM.pdf]

Additional File 14

Cellular composition of malignant ascites samples used in Figures 6 and 7.

Sample ID	Proportion of total cells in sample (%)							
	**CD4**	**CD8**	**EpCAM**	**FAP**	**PD-L1**	**CD11b**	**CD206**	**FR**
Patient 10	35.4	5.95	32.1	29.1	39.2	38.5	23.8	49.0
Patient 12	30.1	7.90	4.80	0.77	-	36.4	19.6	43.4
Patient 14	30.9	10.5	11.5	1.80	-	38.6	37.8	44.8
Patient 15	30.2	9.04	22.6	2.41	24.6	43.2	29.2	31.9
Patient 16	12.2	14.2	5.42	4.0	43.6	43.6	17.6	48.3
