# Supplementary material for: Bi- and tri-valent T cell engagers deplete tumour-associated macrophages in cancer patient samples
Source: J Immunother Cancer. 2019 Nov 21;7:320. doi: 10.1186/s40425-019-0807-6 (PMC6873687; doi:10.1186/s40425-019-0807-6)
Supplement: Supplementary file 15 — Additional file 15. Patient indications for malignant ascites samples used in this study. [file 40425_2019_807_MOESM15_ESM.pdf]

# Additional File 15

Patient indications for malignant ascites samples used in this study.

| Sample ID | Cancer type |
|---|---|
| Patient 1 | Unknown |
| Patient 2 | Ovarian |
| Patient 3 | Ovarian |
| Patient 4 | Ovarian |
| Patient 5 | Melanoma |
| Patient 6 | Oesophageal |
| Patient 7 | Ovarian |
| Patient 8 | Pancreatic |
| Patient 9 | Pancreatic |
| Patient 10 | Breast |
| Patient 11 | Peritoneal |
| Patient 12 | Breast |
| Patient 13 | Serous tubal |
| Patient 14 | Unknown |
| Patient 15 | Ovarian |
| Patient 16 | Breast |
